# Microglia control the spread of neurotropic virus infection via P2Y12 signalling and recruit monocytes through P2Y12-independent mechanisms

**DOI:** 10.1007/s00401-018-1885-0

**Published:** 2018-07-19

**Authors:** Rebeka Fekete, Csaba Cserép, Nikolett Lénárt, Krisztina Tóth, Barbara Orsolits, Bernadett Martinecz, Előd Méhes, Bálint Szabó, Valéria Németh, Balázs Gönci, Beáta Sperlágh, Zsolt Boldogkői, Ágnes Kittel, Mária Baranyi, Szilamér Ferenczi, Krisztina Kovács, Gergely Szalay, Balázs Rózsa, Connor Webb, Gabor G. Kovacs, Tibor Hortobágyi, Brian L. West, Zsuzsanna Környei, Ádám Dénes

**Affiliations:** 1 “Momentum” Laboratory of Neuroimmunology, Institute of Experimental Medicine, Hungarian Academy of Sciences, Szigony u. 43, 1083 Budapest, Hungary; 2 Department of Biological Physics, Eötvös University, Budapest, Hungary; 3 Laboratory of Molecular Pharmacology, Institute of Experimental Medicine, Hungarian Academy of Sciences, Budapest, Hungary; 4 Department of Medical Biology, Faculty of Medicine, University of Szeged, Szeged, Hungary; 5 Laboratory of Molecular Neuroendocrinology, Institute of Experimental Medicine, Hungarian Academy of Sciences, Budapest, Hungary; 6 Laboratory of 3D Functional Network and Dendritic Imaging, Institute of Experimental Medicine, Hungarian Academy of Sciences, Szigony u. 43, 1083 Budapest, Hungary; 7 Institute of Neurology, Medical University of Vienna, Vienna, Austria; 8 Neuropathology and Prion Disease Reference Center, Semmelweis University, Budapest, Hungary; 9 MTA-DE Cerebrovascular and Neurodegenerative Research Group, University of Debrecen, Debrecen, Hungary; 10 Institute of Psychiatry Psychology and Neuroscience, King’s College London, London, UK; 11 Institute of Pathology, Faculty of Medicine, University of Szeged, Szeged, Hungary; 12 Plexxikon Inc, Berkeley, CA 94710 USA

**Keywords:** Microglia, P2Y12, Neurotropic virus, Transsynaptic spread, Neuroinflammation

## Abstract

Neurotropic herpesviruses can establish lifelong infection in humans and contribute to severe diseases including encephalitis and neurodegeneration. However, the mechanisms through which the brain’s immune system recognizes and controls viral infections propagating across synaptically linked neuronal circuits have remained unclear. Using a well-established model of alphaherpesvirus infection that reaches the brain exclusively via retrograde transsynaptic spread from the periphery, and in vivo two-photon imaging combined with high resolution microscopy, we show that microglia are recruited to and isolate infected neurons within hours. Selective elimination of microglia results in a marked increase in the spread of infection and egress of viral particles into the brain parenchyma, which are associated with diverse neurological symptoms. Microglia recruitment and clearance of infected cells require cell-autonomous P2Y12 signalling in microglia, triggered by nucleotides released from affected neurons. In turn, we identify microglia as key contributors to monocyte recruitment into the inflamed brain, which process is largely independent of P2Y12. P2Y12-positive microglia are also recruited to infected neurons in the human brain during viral encephalitis and both microglial responses and leukocyte numbers correlate with the severity of infection. Thus, our data identify a key role for microglial P2Y12 in defence against neurotropic viruses, whilst P2Y12-independent actions of microglia may contribute to neuroinflammation by facilitating monocyte recruitment to the sites of infection.

**Electronic supplementary material:**

The online version of this article (10.1007/s00401-018-1885-0) contains supplementary material, which is available to authorized users.

## Introduction

Microglia are the main immunocompetent cell type of the brain that play a role in diverse physiological processes, including brain development, synaptic plasticity and memory. In turn, alterations in microglial function are linked with common brain diseases such as stroke, epilepsy, Alzheimer’s or Parkinson’s disease [3, 28, 47, 63]. But how microglial actions determine the fate of individual neurons remained largely unclear to date. Microglia are capable of removing synapses via complement-mediated manner [26, 59] and eliminate injured neurons via recognising translocated phosphatidylserine on the cell membrane [7]. Numerous mediators including chemokines, metalloproteinases, growth factors, purinergic metabolites, alarmins or damage-associated molecular patterns such as HMGB1, histones and DNA have been revealed as indicators of neuronal injury and triggers for microglial activation [9, 29, 30, 57, 58]. Among these, microglial P2Y12 receptor-mediated actions have been shown to facilitate the movement of microglial processes to sites of injury or ATP administration [14, 25], whilst constant microglial surveillance of the brain was found to be largely P2Y12-independent [39, 53]. However, whether microglial P2Y12 is involved in early recognition and clearance of injured neurons independently of mediating generic microglial actions to tissue injury remained unclear.

One of the major difficulties in studying microglial responses to mediators released from stressed neurons in vivo is the immediate response of surveilling microglia to any noxious stimuli [46], which is an inherent confounder of most experimental models that include manipulation in the brain parenchyma. To overcome these difficulties, we took advantage of the genetically modified strains of pseudorabies virus (PRV), a member of the subfamily Alphaherpesvirinae (similar to human herpes simplex virus type 1 or varicella-zoster virus), widely used for neuroanatomical tract-tracing and as a well-established model of neurotropic virus infection [6, 8]. When injected into peripheral organs, the “Bartha-Dup” strains of PRV display slow and highly specific retrograde transsynaptic spread in central autonomic circuits [6], allowing us to study directed microglial recruitment in parallel with the propagation of virus infection in the brain using in vivo imaging and advanced histological approaches. Since in this model microglia may only sense signals identifying affected neurons in their vicinity, but do not become infected with PRV even under ex vivo conditions unlike in the case of most viruses with potential or preferential neurotropism in vivo [6, 50], we could also investigate the functional role of microglia and microglial P2Y12 receptors in the control of infection together with the associated neuroinflammatory response.

Although some previous papers have implicated microglia in anti-viral immunity [11, 17, 40, 49, 50], most of them have not used selective microglia manipulation in vivo, and the potential mechanisms of early microglia recruitment including the role of microglial P2Y12 have not been investigated. We have shown earlier that microglia sense changes in neuronal activity and selective elimination of microglia by CSF1R-blockade leads to the dysregulation of neuronal network activity and augmented brain injury [56]. Since neurons infected with PRV-Bartha derivatives have normal electrophysiological characteristics but display increased activity [42], we hypothesised that microglia may detect detrimental changes in the case of individual cells before irreversible neuronal injury occurs. Using this model system, we demonstrate the rapid and targeted recruitment of microglia to compromised neurons using in vivo two-photon and in vitro time-lapse imaging and show that nucleotides released from infected neurons mediate this effect via microglial P2Y12 receptors. We show that through these actions, microglia are instrumental to prevent contact infection and control the transneuronal spread of neurotropic virus infection. Whilst demonstrating the role for P2Y12 in the elimination of infected cells by microglia in both the mouse and the human brain, we also show that microglia, but not microglial P2Y12 contribute to the recruitment of monocytes to transsynaptically infected neurons during viral encephalitis. Since neurotropic herpesviruses are capable of causing both acute and chronic infection in humans and also contribute to diverse forms of neurodegeneration [38, 64], these results are likely to be relevant for a number of neuroinflammatory and neurodegenerative diseases.

## Materials and methods

### Mice

Experiments were carried out on 12–18 weeks old C57BL/6 J, P2Y12^−/−^, P2RX7^−/−^, Cx3Cr1^GFP/+^ and Cx3Cr1^GFP/+^ P2Y12^−/−^ mice. All experimental procedures were in accordance with the guidelines set by the European Communities Council Directive (86/609 EEC) and the Hungarian Act of Animal Care and Experimentation (1998; XXVIII, Sect. 243/1998), approved by the Animal Care and Use Committee of the IEM HAS.

### Selective elimination of microglia from the brain

Mice were fed PLX5622 (Plexxikon Inc. Berkeley, USA; 1200 mg PLX5622 in 1 kg chow) for 3 weeks to eliminate microglia from the brain.

### Neurotropic herpesvirus infection

Mice were randomly assigned to experimental groups and were injected either intraperitoneally or directly into the epididymal white adipose tissue with a genetically modified PRV-Bartha derivative, PRV-Bartha-Dup-Green (BDG) [5] to induce retrograde transsynaptic infection in the brain. In a set of studies, mice were infected with BDG on 16th day of PLX5622 diet to assess the effect of microglia depletion on central propagation of virus infection. For in vivo two-photon imaging, Cx3Cr1^GFP/+^ mice were infected with PRV-Bartha-DupDsRed (BDR) [4] enabling the co-detection of infected neurons with microglia. After virus injection, mice were let to survive for 5–7 days depending on study design and were regularly monitored for neurobehavioral symptoms.

### Tissue processing and immunofluorescence

Brain tissues were fixed by transcardial perfusion (saline, followed by 4% PFA). Immunofluorescence was performed on 25 μm thick free-floating brain sections. Images were captured with a Nikon Ni-E C2 + confocal microscope.

### Super-resolution (STORM) microscopy

Sections were mounted onto #1.5 thick borosilicate coverslips and covered with imaging medium [50] immediately before imaging. STORM imaging was performed for P2Y12 (stimulated by a 647 nm laser) using a Nikon N-STORM C2 + super-resolution system that combines ‘Stochastic Optical Reconstruction Microscopy’ technology and Nikon’s Eclipse Ti research inverted microscope to reach a lateral resolution of 20 nm and axial resolution of 50 nm [1, 18].

### Immuno-electron microscopy

After the combined immunogold–immunoperoxidase stainings, sections were treated with osmium tetroxide, dehydrated in ascending ethanol series and acetonitrile, and embedded in epoxy resin. During dehydration, sections were treated with uranyl acetate. After polymerization, 70 nm thick sections were cut on an ultramicrotome, picked up on formvar-coated single-slot copper grids, and sections were examined using a Hitachi H-7100 electron microscope.

### Correlated confocal laser-scanning microscopy, electron microscopy and electron tomography

Following multiple immunofluorescent staining, sections were analysed using a Nikon Eclipse Ti-E inverted microscope, and an A1R laser confocal system. After imaging, sections were recovered and the immunoperoxidase reaction was developed. Electron tomography was performed using a Tecnai T12 BioTwin electron microscope equipped with a computer-controlled precision stage and an Eagle™ 2 k CCD 4 megapixel TEM CCD camera. Reconstruction was performed using the IMOD software package.

### Post-mortem human brain samples

To investigate microglia recruitment in response to neurotropic virus infection in the human brain, paraffin-embedded (FFPE) post-mortem brain tissues from five patients with known HSV-encephalitis aged 42–66 years were analysed (ethical approval ETT-TUKEB 62031/2015/EKU, 34/2016 and 31443/2011/EKU (518/PI/11)). Tissue samples from two additional patients with no known neurological disease were used as controls. After deparaffinisation, immunohistochemistry was performed, and representative pictures were captured using a Nikon Ni-E C2 + microscope.

### Two-photon imaging

To assess microglia recruitment to infected neurons in the mouse brain in real-time, Cx3CR1^GFP/+^ mice were i.p. injected with 10 μl of the BDR virus. The survival time was set to 7 days post-infection, when numerous infected cells were present in the cerebral cortex. After cranial window preparation, measurements were performed on a Femto2D-DualScanhead microscope (Femtonics Ltd., Hungary) coupled with a Chameleon Ultra II laser [10, 27]. Data acquisition was performed by MES softver (Femtonics Ltd.), two-photon image sequences were exported from MES and analysed using ImageJ.

### Primary neuronal, astroglia and microglia cell cultures

Primary cultures of embryonic cortical cells were prepared from mice on embryonic day 15 [12] and astroglia/microglia mixed cell cultures were prepared from the whole brains of mouse pups, as described earlier [32]. Microglial cells were isolated from 21 to 28 days old mixed cultures either by shaking or by mild trypsinization. In cultures used for time-lapse recordings, microglial cells were seeded on top of astrocytic or neuronal cell cultures in 10000 cell/cm^2^ density. Neuronal or astroglia cultures were infected with either PRV-Bartha-Dup-Green (BDG) virus or PRV-Bartha-DupLac (BDL) at a final titer of 2.5 × 10^5^ PFU/ml, as described earlier [24]. The multiplicity of infection (MOI) was ~ 0,17 PFU/cell.

### Time-lapse microscopy and cell motility data analysis

Time-lapse recordings were performed on a computer-controlled Leica DM IRB inverted fluorescent microscope. Phase contrast and epifluorescent images were collected consecutively every 10 min for up to 48 h post-infection. Images were edited using NIH ImageJ software. Cell tracking: images were analysed individually with the help of custom-made cell-tracking programs (G-track and Wintrack) enabling manual marking of individual cells and recording their position parameters into data files.

### Cytokine measurement from media of primary cell cultures

Concentrations of IL-1α, IL-1β, TNF-α, IL-6, MCP-1, RANTES (CCL5), G-CSF and KC (CXCL1) were measured from conditioned media of primary neuronal, astroglial and microglial cell cultures using cytometric bead array (CBA) Flex Sets. Measurements were performed on a BD FACSVerse machine and data were analysed using an FCAP Array software (BD Biosciences) as described earlier [16]. The cytokine levels of conditional media were corrected for total protein concentrations of the samples measured by Bradford Protein Assay Kit.

### Total RNA isolation and quantitative RT-PCR

For total RNA isolation, cell culture samples were homogenized in 500 μl TRI Reagent and isolation was performed using Tissue Total RNA Mini Kit according to the manufacturer’s instructions. The primers were used in real-time PCR reaction with Fast EvaGreen qPCR Master Mix on a StepOnePlus instrument. The gene expression was analysed using the StepOne 2.3 program. Amplicons were tested by Melt Curve Analysis on StepOnePlus instrument. Experiments were normalized to *gapdh* expression.

### Quantification of nucleotides and adenosine

The adenine nucleotides (ATP, ADP, AMP) and adenosine (Ado) were determined in extracts from cells and culture media using HPLC method. The HPLC system used was a Shimadzu LC-20 AD Analytical & Measuring Instruments System, with an Agilent 1100 Series Variable Wavelength Detector set at 253 nm.

### Immunohistochemical staining for NTPDase1

Coronal brain sections were incubated in the solution of the polyclonal NTPDase1 antibody. After secondary antibody incubation and chromogen development, sections were osmificated, dehydrated in ascending ethanol series, and embedded in Taab 812 resin. Ultrathin sections were examined using a Hitachi 7100 transmission electron microscope.

### Enzyme histochemistry for detection of ecto-ATPase activity

A cerium precipitation method was used for electron microscopic investigation of ecto-ATPase activity [31]. The tissue blocks were then postfixed, dehydrated, treated and embedded into Taab 812 resin for ultrathin sectioning and microscopic examination.

### Flow cytometric analysis of brain, spleen and blood samples

Cells were isolated from mouse brains by enzymatic digestion with the mixture of DNase I and Collagenase/Dispase. Spleen cells were isolated by mechanical homogenization of the spleen. Venous blood was collected from the heart before transcardial perfusion using 3.8% sodium citrate as an anticoagulant. Cells were acquired on a BD FACSVerse flow cytometer and data were analysed using FACSuite software. Total blood cell counts were calculated using 15 μm polystyrene microbeads.

### Statistical assessment

All quantitative measurements and analysis were performed in a blinded manner in accordance with STAIR and ARRIVE guidelines. Data were analysed using the GraphPad Prism 7.0 software. For comparing two experimental groups Student’s *t* test with Welch’s correction or Mann–Whitney U test, for comparing three or more groups one-way or two-way ANOVA followed by Tukey’s, Sidak’s and Dunnett’s post hoc comparison was used. *P* < 0.05 was considered statistically significant.

Please refer to the Supplementary Methods (Online Resource 1) for additional details.

## Results

### Microglia are instrumental for anti-viral immunity in the brain

**Fig. 1:**
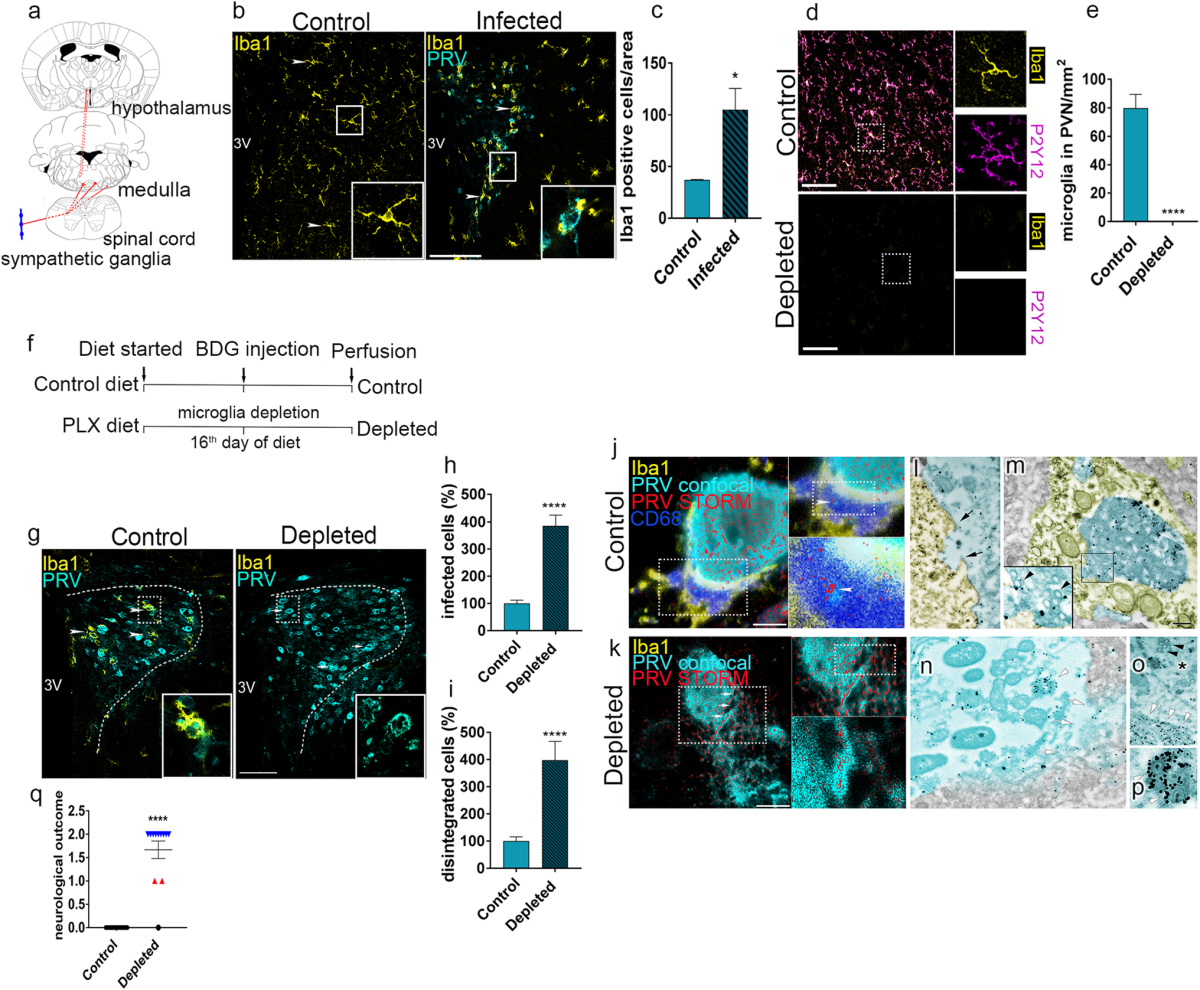
Selective elimination of microglia results in uncontrolled viral spread and neurobehavioral pathologies. **a** Retrograde transneuronal spread of PRV-Bartha-DupGreen (BDG) to the hypothalamic paraventricular nucleus (PVN) 6 days after intraperitoneal injection. **b** Focal neuronal infection induces recruitment of microglia (Iba1: yellow) to infected neurons (PRV: cyan) in the PVN. **c** Microglial numbers increase significantly at sites of virus-infected neurons, area: 0.2 mm^2^. **d** Selective depletion of microglia by PLX5622 for 3 weeks (yellow: Iba-1, magenta: P2Y12). **e** Depletion results in the elimination of 96% of microglia from the PVN. **f** An absence of microglia results in profound increases in virus-infected neurons in the brain. Control and microglia-depleted mice were injected intraperitoneally with BDG on the 16th day of the diet and allowed to survive for 5 days to induce transsynaptic spread of PRV to the hypothalamus. Note the numerous infected neurons outside the nucleus indicating increases in non-synaptic (contact) infection in the absence of microglia. **g** The number of infected neurons in the PVN increases over twofold in microglia depleted mice 5 days after virus injection. **h** An absence of microglia leads to profound increases in the number of infected neurons in the PVN. **i** An absence of microglia leads to increased number of infected, disintegrated neurons in the PVN. **j** Superresolution microscopic analysis (STORM) reveals the colocalization of the phagosome marker, CD68 (dark blue), with neuronal debris containing viral structural proteins (PRV) inside recruited microglia. **k** An absence of microglia leads to massive increases in extracellular virus proteins around infected, disintegrated neurons. **l–p** Transmission electron microscopic (TEM) images showing infected neurons in microglia competent (**l**, **m**) and depleted (**n**–**p**) animals. **l** Microglial processes (yellow pseudocolor) are found tightly attached to the cell membrane of infected neuron (cyan pseudocolor), as identified by anti-PRV immunogold labelling (black grains). Black arrows indicate a disrupted neuronal membrane segment isolated by a microglial process. **m** Infected neuronal cell debris is phagocytosed by microglia. Note the presence of viral capsids inside phagocytosed neuronal particles labelled with anti-PRV immunogold (insert). **n** In the absence of microglia disintegrated neuronal membranes with anti-PRV-immunogold labelled content invading the surrounding neuropil can be seen (white arrows). **o** Infected neuron contains viral capsids (black arrowheads), asterisk marks the nucleus, mature virions with strong PRV-immunogold labelling can be seen in the cytoplasm (white arrowheads). **p** PRV-virions are displayed at higher magnification. **q** Absence of microglia results in rapidly appearing neurological symptoms 5 days after PRV infection (0: no symptoms, 1: drooling and heavy breathing, 2: seizures and muscle spasms). Data expressed as mean ± s.e.m c *p < 0.05 *n* = 4–5, Mann–Whitney test **e** ****p < 0.0001 *n* = 6 unpaired *t* test, **h**, **i** ****p < 0.0001 Mann–Whitney test *n* = 10–11 **i** **p < 0.001 unpaired *t* test *n* = 9–12, q ****p < 0.0001 Mann–Whitney test *n* = 20–21 Scale bars: **b** 50 µm, **d** 50 µm, **g**, 100 µm, **j**–**k** 100 µm. Scale bar on **m** is 400 nm for **l**–**o**, 175 nm for *p*

To study whether microglia respond to local cues and are recruited to virus-infected neurons, we made use of the precisely controlled, retrograde transsynaptic spread of the PRV derivative, Bartha-DupGreen (BDG) to central autonomic nuclei [5, 6] from peripheral targets (Fig. 1a). In the hypothalamic paraventricular nucleus (PVN), microglial numbers increased threefold in response to infection and infected neurons were surrounded by numerous Iba1-positive cells (Fig. 1b, c), 6 days after intraperitoneal (i.p.) virus injection. To investigate whether microglia are involved in the control of neurotropic virus infection, we performed selective depletion of microglia, by feeding mice the CSF1R antagonist PLX5622, as demonstrated earlier [20, 56]. After 3 weeks of depletion, 96% of microglia were eliminated from the brain as evidenced by the lack of the microglial markers Iba1 and P2Y12 (Fig. 1d, e). Selective elimination of microglia resulted in a marked increase in the number of virus-infected neurons in the brain (Fig. 1f–g). This phenomenon was not dependent on the route of virus administration, since after i.p. virus injection or injection of the virus into the epididymal white adipose tissue (an organ receiving predominantly sympathetic innervation [2]), the number of virus-infected neurons was much higher in the brain in the absence of microglia [Fig. 1h; Suppl. Fig. 1 (Online Resource 1)]. In microglia-depleted mice, numerous infected cells were also present in the cerebral cortex already on day 5, and retrograde infection reaching the cortex was more widespread, affecting several areas normally not infected when microglia were present. In addition, more than a threefold increase in the number of disintegrated neurons containing viral proteins was found in the brain parenchyma in microglia-depleted mice (Fig. 1i), indicating the lack of effective elimination of the infected neurons by microglia. To investigate this phenomenon further, we visualized viral proteins using super-resolution microscopy together with the microglial phagosome/lysosome marker CD68 (Fig. 1j, k). In control mice, microglial processes tightly surrounded the cell bodies of infected neurons with viral proteins appearing in microglial phagosomes (Fig. 1j). Importantly, the absence of microglia resulted in a massive increase in extracellular viral proteins and PRV-immunopositive cell debris (Fig. 1k). Confirming these observations, electron microscopy revealed a direct contact between microglial processes and the cell membrane of the infected neurons as well as the uptake of infected neurons by microglia (Fig. 1l, m). In contrast, disintegrated neuronal membranes and extracellular immunogold-labelled viral proteins were observed in microglia-depleted animals (Fig. 1n–p). BDG products, including GFP signal [5, 6], viral capsids and PRV-immunopositive profiles were not observed in the nucleus or the cytoplasm of microglia [Suppl. Fig. 2 (Online Resource 1)], indicating that productive infection does not develop in these cells, in line with earlier reports [6, 50]. The absence of microglia was also associated with the development of diverse neurological symptoms in infected mice starting on the 5th day of infection, when infected neurons were numerous in the brain stem, the hypothalamus and the autonomic-associated nuclei in the limbic system. These symptoms included heavy breathing, muscle spasms and seizure-like episodes, which were absent in control mice (Fig. 1q).

### Microglia recruitment is initiated rapidly to virus-infected neurons in the brain

**Fig. 2:**
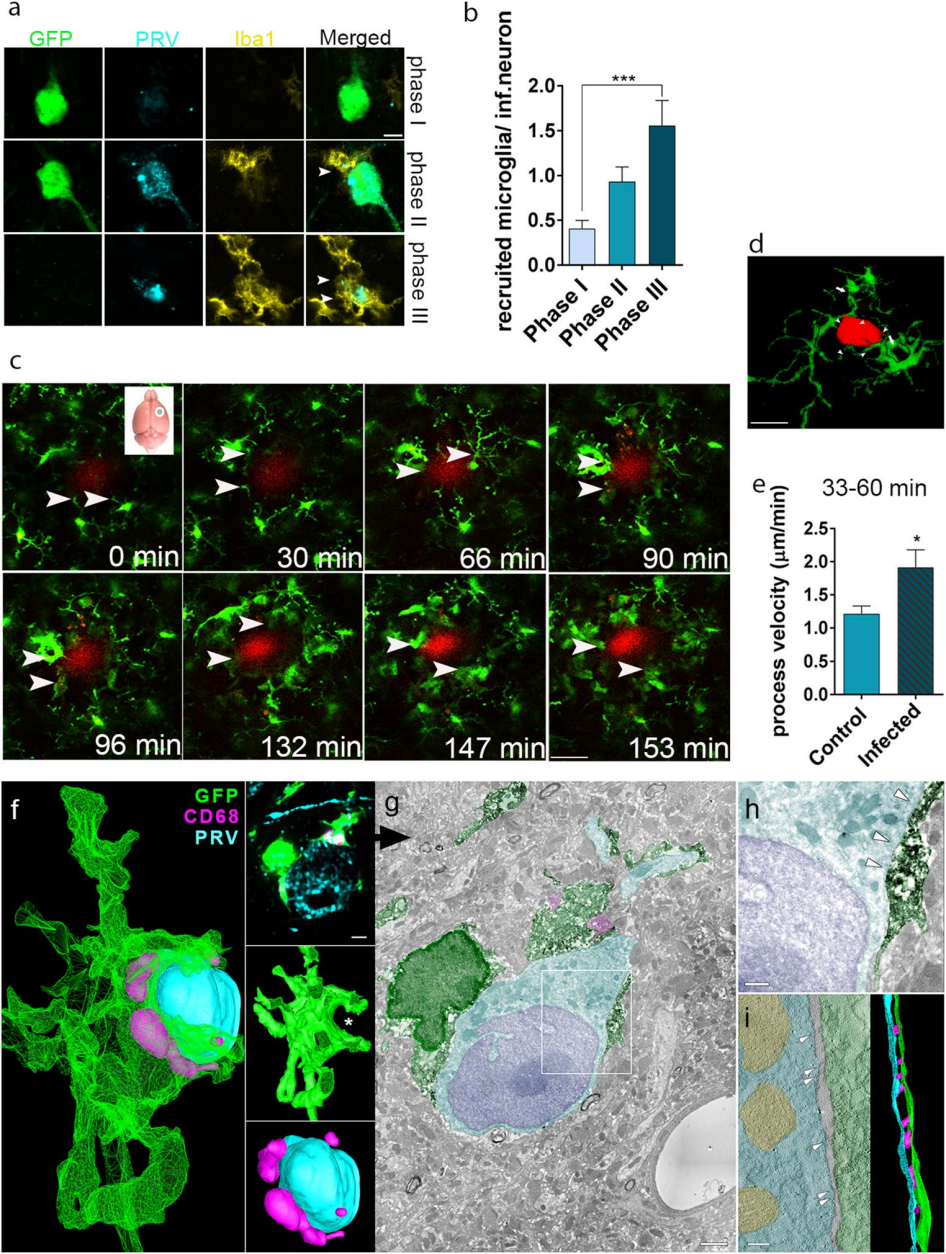
Microglia rapidly isolate virus-infected neurons in the brain. **a** Microglia (Iba1, yellow) are recruited to neurons infected with PRV expressing GFP with immediate-early kinetics (BDG) in the hypothalamic paraventricular nucleus (PVN). Note that microglia recruitment starts after the expression of the immediate-early marker GFP (phase I), when low levels of viral structural proteins become detectable (phase II). High levels of viral structural proteins indicate a late stage of viral infection (phase III), which is associated with higher number of microglia recruited to infected cells. **b** Microglial numbers increase significantly around virus-infected neurons in parallel with the propagation of infection **c** In vivo two-photon imaging reveals the recruitment of microglia (green) to virus-infected neurons (red) in real-time. Retrograde transsynaptic infection was induced by the virus BDR in Cx3Cr1^+/GFP^ microglia reporter mice 7 days prior to imaging to visualize infected cells in the cerebral cortex based on the immediate-early DSRed expression. **d** Merged *Z*-planes of microglial cells around a PRV-DSRed-positive neuron (arrows indicate recruited microglia, arrowheads indicate microglial processes contacting the infected cell). **e** Microglial process velocity increases significantly in response to viral infection compared to that seen in control mice. **f**–**i** Correlated CLSM, electron microscopy and electron tomography confirms direct microglia–neuron contact with intercellular molecular links in early phase of viral infection. **f**, 3D-reconstruction from deconvolved confocal stack of a recruited microglia (green) engulfing a PRV-positive neuronal cell body (cyan) with CD68-positive phagolysosomes (magenta) within the microglia, arranged around infected neuron. Upper insert: single image plane of the same confocal stack. Middle insert: 3D-reconstruction of the same microglial cell, rotated 180° around the vertical axis. Asterisk labels the bay engulfing the soma of the infected neuron. Lower insert: 3D-reconstruction of the infected neuronal cell body with surrounding phagolysosomes located within the microglia. **g** Transmission electron micrograph shows the same cells on an ultrathin section matching the confocal image plane in the upper insert in **f**. Note that microglial processes surround the apical dendrites of the infected neuron. **h** Part of **g** (in white box) enlarged. Note that the nucleus is void of viral capsids and the membrane of the neuron is intact (white arrowheads), confirming the early phase of infection. **i** 3 nm thick electron tomographic section and corresponding 3D-reconstruction shows the very close cell–cell contact between the same microglia (green pseudocolor) and infected neuron (cyan pseudocolor, mitochondria are in yellow). White arrows point to putative contact sites, where the distance between the membranes is the smallest, and several filament-like structures (magenta) can be observed connecting the two cells. **b** ***p < 0.001 one-way ANOVA, *n* = 8; **e** *p < 0.05, unpaired *t* test, *n* = 10. Scale bars: **a** 10 µm; **c** 20 µm; **d** 20 µm; **f** 2 µm; **g** 1 µm; **h** 400 nm; **i** 100 nm

Having confirmed the instrumental role of microglia in controlling neurotropic virus infection, we aimed to investigate whether the recruitment of microglia occurs early enough to allow the isolation of infected neurons prior to the breakdown of neuronal cell membranes. To this end, we made use of the immediate-early marker, GFP, expressed in infected neurons several hours prior to the production of viral structural proteins, which allows time-mapping the different phases of infection at a single neuron level [5, 6, 15]. GFP-positive neurons expressing low levels of viral structural proteins (Phase II cells) already appeared more surrounded by microglia than the majority of neurons expressing GFP only (Phase I, Fig. 2a, b), suggesting that recruitment of microglia is induced within a few hours of infection, by the time viral structural proteins are produced [5, 6, 15]. The number of microglia increased further around neurons expressing high levels of viral proteins (Phase III), resulting in 1–3 microglial cells contacting the cell body of a single, infected neuron (Fig. 2a, b).

To investigate the processes of microglia recruitment in vivo in real-time, we used another virus strain, PRV-Bartha-DupDSRed (BDR), enabling early phases of infection to be identified based on the production of the red fluorescent protein, DSRed [4]. Mice with functional microglia were allowed a longer, 7 days survival after virus injection, resulting in the spread of infection to the upper layers of the cerebral cortex (Fig. 2c). In vivo two-photon imaging in Cx3Cr1^+/GFP^ (microglia reporter) mice revealed the recruitment of microglia within 3 h of the increases observed in neuronal DSRed signal [Suppl. Video 1. (Online Resource 2)]. We used an optimized cranial window preparation for these studies as developed earlier, to avoid any disturbance of microglia [56]. 3D reconstruction from 2P Z-stack revealed that microglial processes formed a barrier-like structure, with several contact points around the cell body of the infected neuron (Fig. 2d). Microglia recruited to infected cells showed increased process velocity compared to microglia distant from sites of virus infection (Fig. 2e), indicating that microglia may respond to local signals induced by infected neurons. To further explore whether microglial contacts with the cell membranes of infected neurons can be formed in the early phases of virus infection, we visualized microglia–neuron contacts with confocal microscopy in Cx3Cr1^+/GFP^ mice, followed by the investigation of selected neurons with correlated electron microscopy and electron tomography. 3D reconstruction from confocal Z-stack revealed the formation of microglial contacts around the cell body and the main dendrites of infected neurons [Fig. 2f; Suppl. Video 2. (Online Resource 3)] prior to the appearance of mature virions in the neuronal cytoplasm (Fig. 2g, h). At this stage of infection, neuronal cell membranes were intact with normal chromatin structure seen in the nucleus [Suppl. Fig. 2b (Online Resource 1)]. Microglial processes surrounding infected neurons showed CD68-immunopositivity, indicating the phagocytic activity of microglia (Fig. 2f, g). In addition, electron tomography revealed the formation of specific membrane interactions between infected neurons and microglia suggesting the recognition and contact of the intact cell membranes by recruited microglial processes (Fig. 2i).

### Virus-infected cells are recognised and engulfed by microglia in vitro

**Fig. 3:**
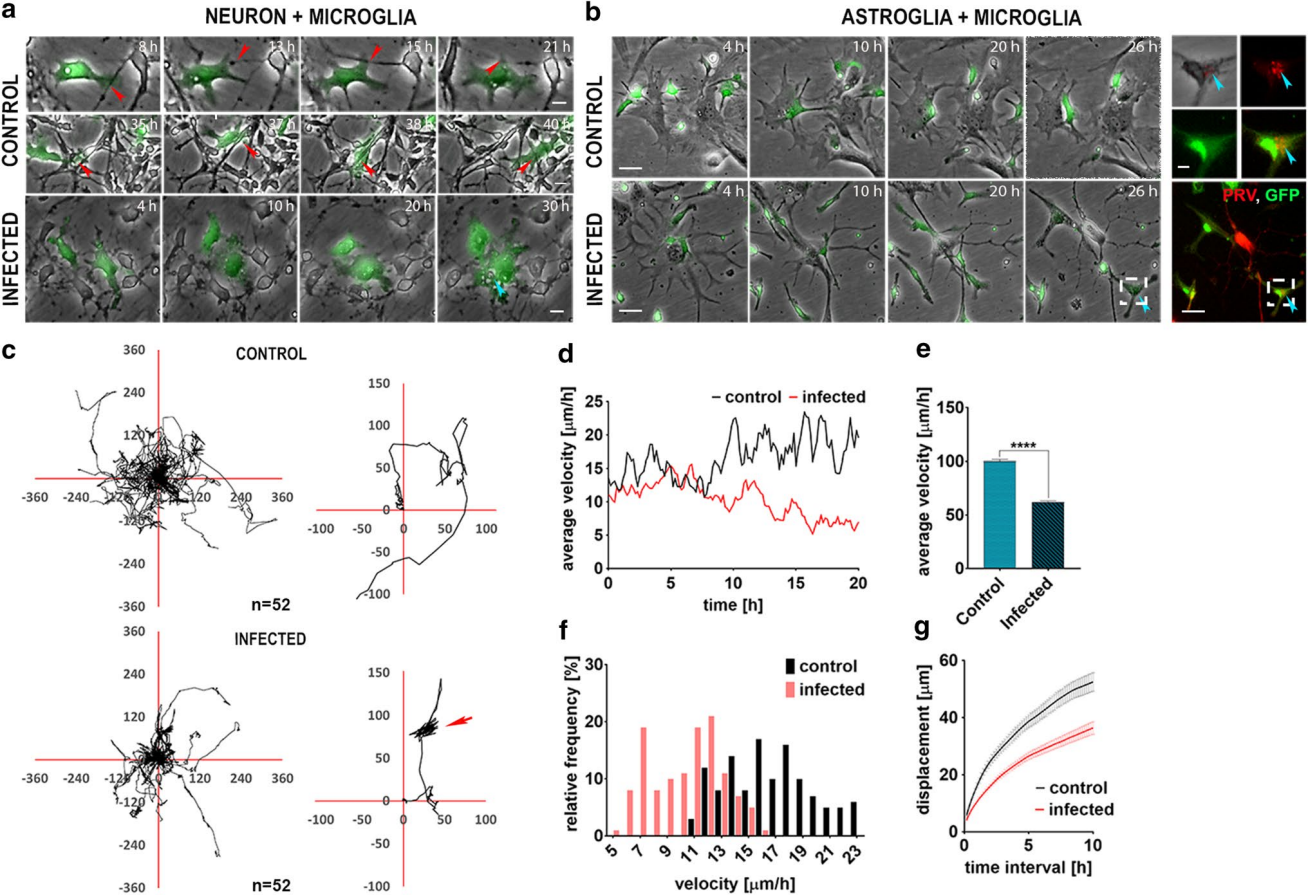
Virus infection triggers the recruitment and phagocytic activity of microglia in vitro. **a** Cx3Cr1^+/gfp^ microglial cells (green) repeatedly interact with non-infected neuronal processes and cell bodies without hindering process outgrowth or affecting network stability. Red arrows point to an uninterruptedly growing neurite (upper panel and Supplementary Video 3.) or a microglia slipping under a dense neuronal network (mid panel and Supplementary Video 4.). Microglial cells contacting PRV-infected neurons flatten and phagocytose compromised cells (blue arrow, bottom panel), also see Supplementary Video 5. **b** Cx3Cr1^+/gfp^ microglial cells (green) move freely in non-infected astroglial cultures making and releasing contacts with several astrocytes (upper panel and Supplementary Video 6.) while they are recruited to infected cells (bottom panel and Supplementary Video 7.) and after scanning they display phagocytic activity (blue arrow). Satellite images: PRV + particles (red), eaten up by a Cx3Cr1^+/gfp^ microglial cell (green). **c** Trajectories of randomly chosen microglial cells (*n* = 52) over 24 h in control or infected astroglial cultures. Individual cell trajectories were centered to start from the origin and superimposed for better comparison of migration directionality. Insets show typical trajectories. Note the random walk behaviour in the trajectory in the upper inset (control), and the shorter trajectory with targeted directionality (arrow) characterizing the slower migrating and scanning microglial cells in infected cultures in the lower inset. **d** Time-dependent average velocities of microglial cell populations in control (*n* = 54) or infected (*n* = 56) astroglial cultures. **e** Average velocities of microglial cells over 24 h are significantly lower in infected astroglial cultures. **f** Frequency distribution of time-dependent average velocities of microglial populations in control or infected astroglial cultures. **g** Average displacement of microglial cells in various time intervals of migration in control or infected astroglial cultures. Error stripes correspond to s.e.m. Scale bars: **a** 10 µm; **b** 25 and 10 µm, respectively. Data are expressed as mean ± s.e.m. **e** *n* = 121, unpaired *t* test, ****p < 0.0001

To study the mechanisms of microglia recruitment to sites of infection, we first established co-cultures of neurons and GFP-positive microglia from Cx3Cr1^+/GFP^ mice and performed time-lapse imaging over a 48 h period. Microglia contacted the cell body and the main processes of uninfected neurons without causing injury or showing phagocytic activity [Fig. 3a, upper and mid panel and Suppl. Videos 3, 4. (Online Resources 4, 5)]. In contrast, microglia added to virus-infected neurons were recruited to and phagocytosed infected cells [Fig. 3a, bottom panel and Suppl. Video 5. (Online Resource 6)]. Next, we aimed to study the behaviour of microglia with more advanced statistical approaches [24, 43, 44], which required co-cultures of microglia with very sparsely distributed cells. Since this was not feasible with neuronal cultures, we took advantage that astrocytes are also infected with PRV under in vitro conditions [24]. In fact, in sparse astrocyte cultures microglial cells migrated much longer distances on average to reach infected cells. Statistical analysis of longer cell trajectories thus enabled us to more effectively separate random migration from targeted migration of microglial cells to infected cells, followed by localized scanning activity and phagocytosis [Fig. 3b, upper panel and Suppl. Video 6. (Online Resource 7)].

As seen in neuronal cultures, microglia recognised and phagocytosed infected astrocytes, which was confirmed by immunofluorescent detection of the engulfed cells after imaging [Fig. 3b, bottom panel and insert, Suppl. Video 7. (Online Resource 8)]. Statistical analysis revealed the recruitment of microglia to PRV-infected cells and the formation of prolonged cell-to-cell contacts. This was evidenced by microglia trajectories showing characteristic localized pattern as cells tend to remain at virus-infected cells once they met them (Fig. 3c), which is in sharp contrast with microglia trajectories in uninfected cultures showing a random walk behaviour pattern. This phenomenon was associated with a reduction of cell velocities in infected cultures (16.6 ± 3.2 µm/h in control and 10.3 ± 2.6 µm/h in infected cultures, Fig. 3d–g) indicating that signals from infected cells direct microglial migration, scanning behaviour and subsequent phagocytic activity. Similar microglial responses were seen in neuronal/microglial co-cultures [Suppl. Fig. 3 (Online Resource 1)]. Importantly, the development of productive infection was never observed in microglia in vivo or in vitro even after the direct exposure of the cells to high viral titres or following extensive phagocytic activity, as evidenced by the absence of the immediate-early GFP signal and PRV proteins from microglia outside phagosomes [Suppl. Fig. 4 (Online Resource 1)].

### Nucleotides released from infected cells trigger microglia recruitment and phagocytosis via microglial P2Y12

To investigate the production of inflammatory mediators induced by neurotropic virus infection, we measured several inflammatory cytokines and chemokines that are commonly upregulated in response to virus infection [37] in cultured neurons and astrocytes. Bacterial lipopolysaccharide (LPS), a widely used proinflammatory stimulus, induced a robust increase in TNFα, IL-6, CXCL1, CCL5 (RANTES), G-CSF and MCP-1 levels in astrocytes and CXCL1, CCL5 and MCP-1 levels in neurons. In contrast, virus infection increased only CCL5 levels in both cell types at mRNA and peptide levels 24 h after infection [Suppl. Fig. 5 (Online Resource 1)], which did not explain the rapid recruitment of microglia to infected cells.

**Fig. 4:**
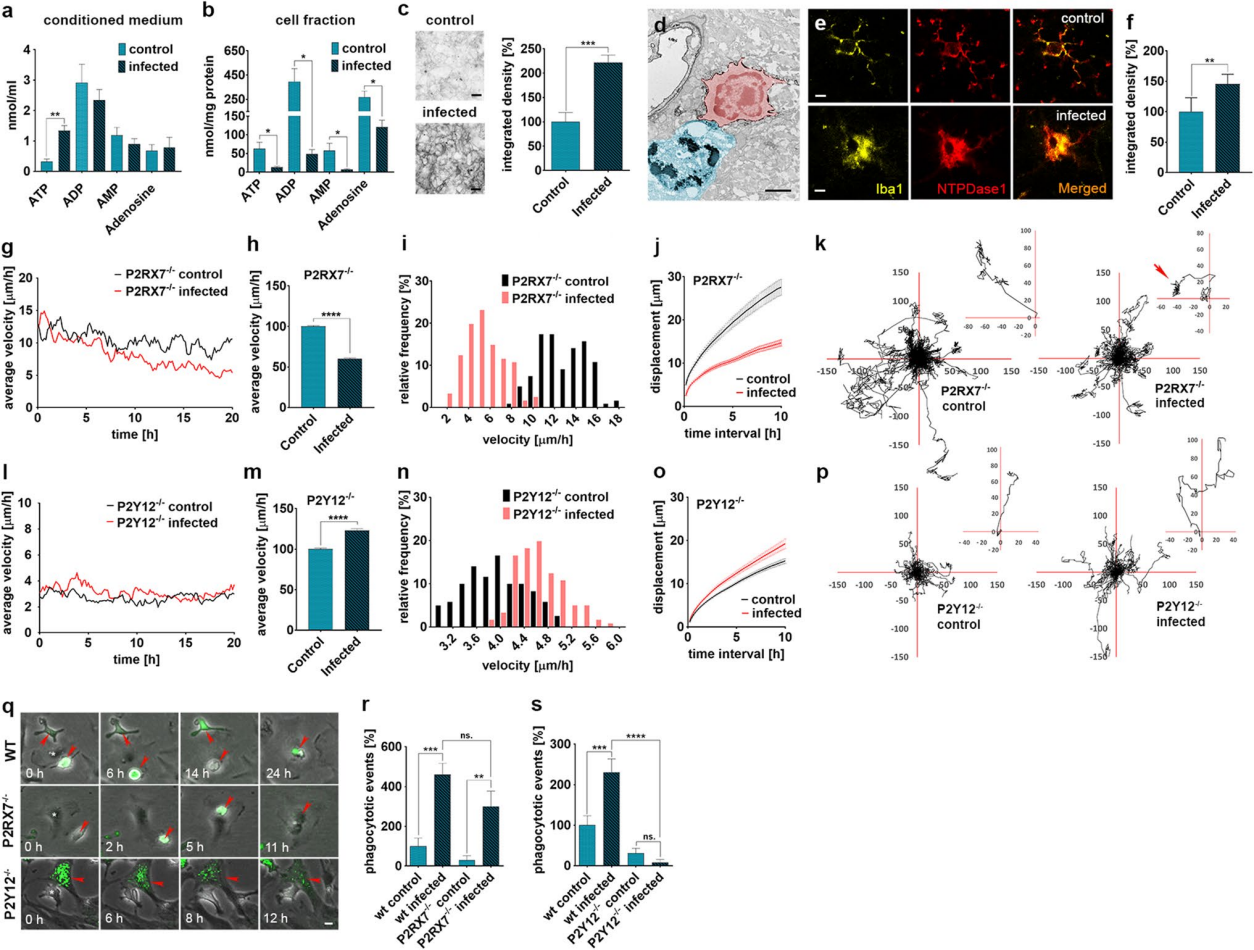
Purinergic signaling mediates microglia recruitment and phagocytosis upon virus infection. **a** and **b**, HPLC analysis of ATP, ADP, AMP and adenosine levels in the supernatant (**a**) and in the cellular fraction (**b**) of control and virus-infected neuronal cultures. **c** Ecto-ATPase enzyme histochemistry and densitometric analysis. The ecto-ATPase activity is significantly enhanced upon infection. **d** NTDPase1 expression of a microglial cell (red) in the PRV-infected brain. Note that the NTDPase1^+^ microglia is in contact with an apparently disintegrating neuron (blue). **e** NTDPase1 expressing Iba1 + microglial cells in control and infected mouse brains. **f** Densitometric analysis of NTDPase1 expression in control and infected mouse brain slices. **g** and **l** Time-dependent average velocities of P2X7^−/−^ (*n*_c_ = 67, *n*_i_ = 74) or P2Y12^−/−^ (*n*_c_ = 51, *n*_i_ = 59) microglial cell populations in control or infected astroglial cultures. Note the gradual decrease in population velocity of P2X7^−/−^ microglia in infected culture and the lack of such decrease in P2Y12^−/−^ microglia in similar conditions. **h** and **m** Average velocities of P2X7^−/−^ or P2Y12^−/−^ microglial cells over 24 h in control or infected astroglial cultures, **i** and **n** Frequency distribution of time-dependent average velocities of P2X7^−/−^ or P2Y12^−/−^ microglial populations in control or infected astroglial cultures. **j** and **o** Average displacement of P2X7^−/−^ or P2Y12^−/−^ microglial cells in various time intervals of migration in control or infected astroglial cultures. Error stripes correspond to s.e.m. **k** and **p** Trajectories of equal number (*n* = 50) of randomly chosen P2X7^−/−^ or P2Y12^−/−^ microglial cells over 24 h in control or infected astroglial cultures. Individual cell trajectories were centered to start from the origin and superimposed for better comparison of migration directionality. Insets show typical trajectories. Note the more localized migration pattern due to the scanning activity of P2X7^−/−^ microglia in infected conditions (red arrow) as compared to random walk behaviour and the lack of such localization in the trajectories of P2Y12^−/−^ microglia in infected astroglial culture. **q** Phagocytic activity of wild type (WT), P2X7^−/−^ or P2Y12^−/−^ microglia cells in astroglial cultures infected with BDG virus. Green colour indicates the virus initiated expression of GFP in infected cells. Red arrows point to the phagocytosed cells. Note, that P2Y12^−/−^ microglia is unable to incorporate the infected cell. **r** and **s** Percentage of phagocytic events by wild type vs. P2X7^−/−^ (**r**) or P2Y12^−/−^ (**s**) microglial cells in control and infected cultures. **a**, **b** *n* = 4 per group, unpaired *t* test, *p < 0.05; **p < 0.001 **c** *n* = 6, unpaired *t* test, ***p ≤ 0.0001 **f** *n* = 7, unpaired *t* test, **p < 0.01. **h** and **m** *n* = 121, unpaired *t* test, ****p < 0.0001. **r** and **s,** *n* = 9 per group, one-way ANOVA, **p < 0.001; ***p < 0.0005, ****p < 0.0001, ns = not significant. Scale bars: **c** 25 μm; **d** 5 μm; **e** 10 μm; **q** 10 μm data are expressed as mean ± s.e.m

Since synthesis and release of chemokines could last for several hours and our in vivo data suggested rapid microglia recruitment to sites of virus infection, we checked whether purine nucleotides such as ATP that are chemotactic for microglia at a short time scale [14] could be released from compromised cells. We found that cultured neurons released ATP after virus infection, which was associated with reduced ATP, ADP, AMP and adenosine levels in cell lysates (Fig. 4a, b), within hours upon the expression of the immediate-early marker, GFP, which precedes the expression of viral structural proteins required for productive infection [5, 6, 15]. The changes in purinergic metabolites were associated with increased ecto-ATPase levels in infected cells (Fig. 4c), but were not due to apoptosis or necrosis, since at the early stages of infection neurons expressing high levels of GFP showed no annexin V binding or uptake of propidium iodide [Suppl. Fig. 6 (Online Resource 1)]. In addition, increased ecto-ATPase levels and NTDPase1 expression were found in microglia at sites of virus infection in the brain (Fig. 4d–f), indicating that microglia respond to changes in the levels of purine nucleotides [55]. To investigate the mechanisms mediating microglial responses to purine nucleotides released from infected cells, we assessed microglial responses in co-cultures of P2X7^−/−^ or P2Y12^−/−^ microglia and wild type astrocytes. Similarly to that seen in wild type microglia (Fig. 3c–g), motility of P2X7^−/−^ cells decreased when exposed to infected cells (Fig. 4g–j) and trajectories showed characteristic localized pattern due to frequent scanning activity [Fig. 4k, Suppl. Video 8. (Online Resource 9)], indicating that P2X7 deficiency does not prevent the recognition of virus-infected cells by microglia. In contrast, virus-exposed P2Y12-deficient microglia showed increased motility (Fig. 4l–o) with trajectories characteristic of random walk behaviour and lacking the localized pattern [Fig. 4p, Suppl. Video 9. (Online Resource 10)], suggesting that these cells are unable to display targeted recruitment in response to infection. Furthermore, wild type and P2X7^−/−^ microglia showed a markedly increased phagocytic activity in infected cultures, which was fully abolished in P2Y12-deficient microglia [Fig. 4q–s and Suppl. Videos 10–11 (Online Resources 11–12)]. Thus, P2Y12 is a key contributor to recognition of compromised cells by microglia and to microglial phagocytosis of virus-infected cells in vitro.

### Recruitment of microglia and elimination of virus-infected neurons are mediated by microglial P2Y12 in vivo

**Fig. 5:**
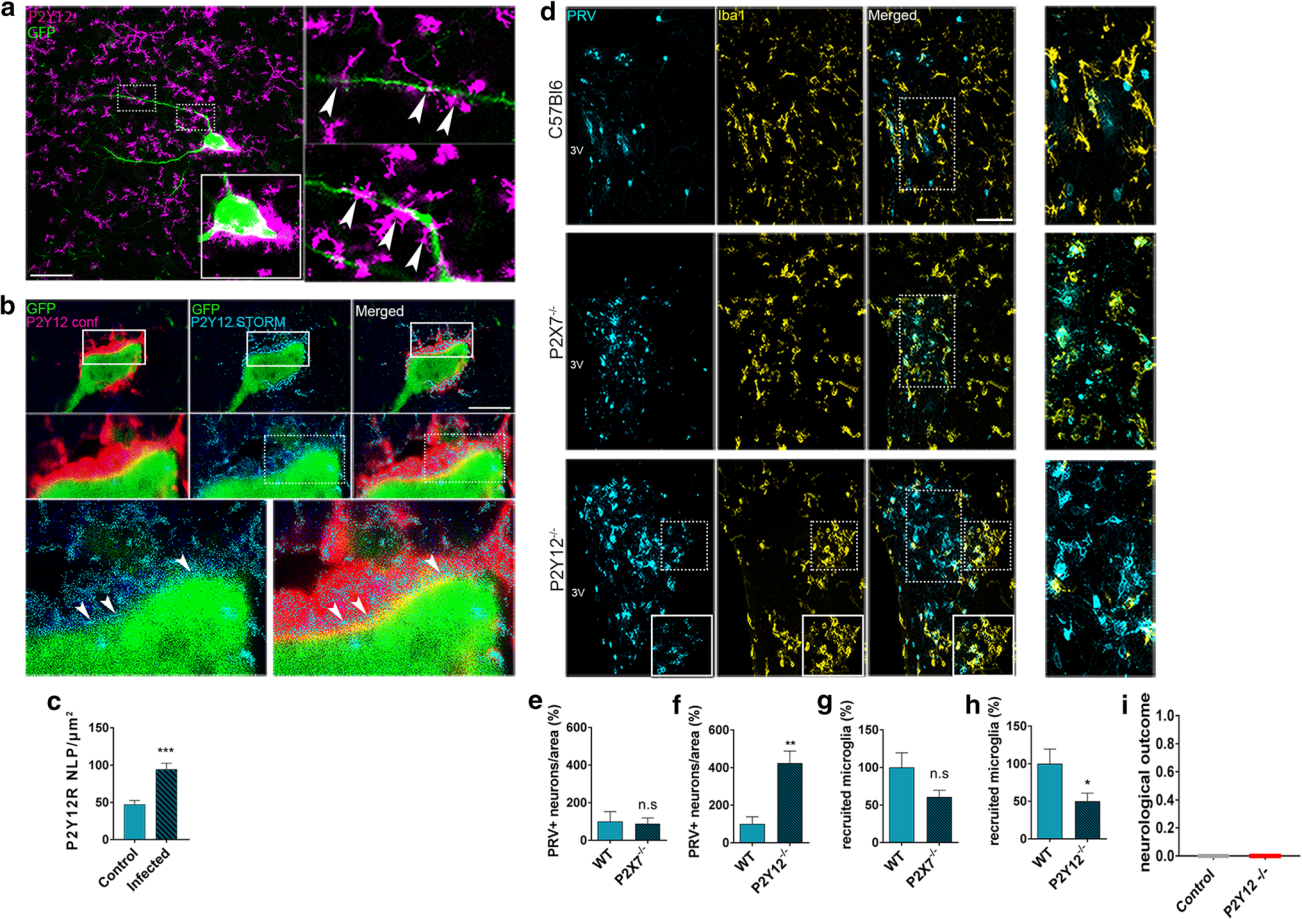
P2Y12 receptor mediates recruitment of microglia in response to virus infection in vivo. **a** P2Y12-positive microglia are recruited to virus-infected neurons in the mouse brain (paraventricular hypothalamic nucleus is shown, maximum intensity projection from confocal Z stack of 30 steps, made with 0,50 µm step size). **b** STORM super-resolution microscopy reveals P2Y12 receptors clustering at microglial cell membranes contacting virus-infected neurons. Neurons are identified by GFP expression with immediate-early kinetics indicating early stages of PRV infection. **c** Based on STORM images, microglial P2Y12 localisation point numbers (NLP) are significantly increased at microglia–neuron contact sites, when microglia contacting infected neurons are compared with microglia contacting uninfected cells. **d** Fluorescent images showing the propagation of virus infection in the paraventricular nucleus of the hypothalamus in wild type, P2X7^−/−^ and P2Y12^−/−^ mice. Infected neurons in the late phase of PRV infection are revealed by immunostaining against viral structural proteins (cyan blue), and microglia are labelled with Iba1 (yellow) (Maximum Intensity Projection from confocal Z stack of 16 steps, made with 0.88 µm step size). **e**, **f** In P2Y12 ^−/−^ mice (*n* = 7) significantly less (*p* < 0.05) microglia are recruited to infected neurons compared to that seen in wild type (*n* = 6) and P2X7^−/−^ mice (*n* = 7), area: 0.2 mm^2^. **g**, **h** Correspondingly, significantly higher numbers of infected neurons are seen in P2Y12^−/−^ mice compared to wild-type animals, whilst no significant difference is observed between P2X7^−/−^ and wild-type mice. area:0,2 mm^2^. **i** Absence of P2Y12 receptor did not result in any neurological symptoms 6 days after virus infection. 3 V - 3rd ventricle. Scale bars: a, 50 µm; b, 100 µm; d, 50 µm. All data expressed as mean ± s.e.m **c ** ***p < 0.0001 unpaired *t* test *n* = 7–13 cells **e**, **g** N.S. not significant **f** **p < 0.01 Mann–Whitney test, *n* = 7 **h** *p < 0.05 *n* = 7 unpaired *t* test

Next, we investigated whether nucleotides released from compromised neurons are involved in the recruitment of microglia in vivo. We found that virtually all microglia surrounding the cell body and the processes of infected neurons in either C57BL/6 or Cx3Cr1^+/GFP^ mice expressed P2Y12 receptors [Fig. 5a, Suppl. Fig. 7 (Online Resource 1)]. STORM super-resolution microscopy, which allowed visualization of P2Y12 receptors at 20 nm lateral resolution showed that microglial P2Y12 receptor numbers increased over twofold in response to infection and P2Y12 clusters in microglial processes contacting infected neurons were localized around the membrane of the infected cell (Fig. 5b, c). To investigate the contribution of purinergic signalling to antiviral immunity in vivo, we induced virus infection in mice lacking P2X7 or P2Y12 receptors (Fig. 5d). Both receptors are abundant in microglia, whereas P2Y12 is a microglia-specific marker in the brain [55] [see also Supp. Fig. 7 (Online Resource 1)]. We found that an absence of P2Y12 resulted in > 50% reduction in the numbers of microglia recruited to infected neurons in the PVN (Fig. 5f, h; p < 0.05), whereas a non-significant trend to reduction (by 35%) was seen in P2X7^−/−^ mice (Fig. 5e, g). Similarly to that seen after selective elimination of microglia (Fig. 1g), the number of infected neurons containing viral structural proteins increased over threefold in P2Y12^−/−^ mice (Fig. 5f, h), but no changes were seen in P2X7^−/−^ mice (Fig. 5e, g). Interestingly, clusters of microglia observed in the brain at sites of virus infection in P2Y12^−/−^ mice were located in the close vicinity of degenerated, PRV-immunopositive neurons, suggesting that P2Y12 deficiency markedly impairs microglial responses to signals released from infected neurons and compromises phagocytic responses, but does not fully block microglial migration to already disintegrated cells.

**Fig. 6:**
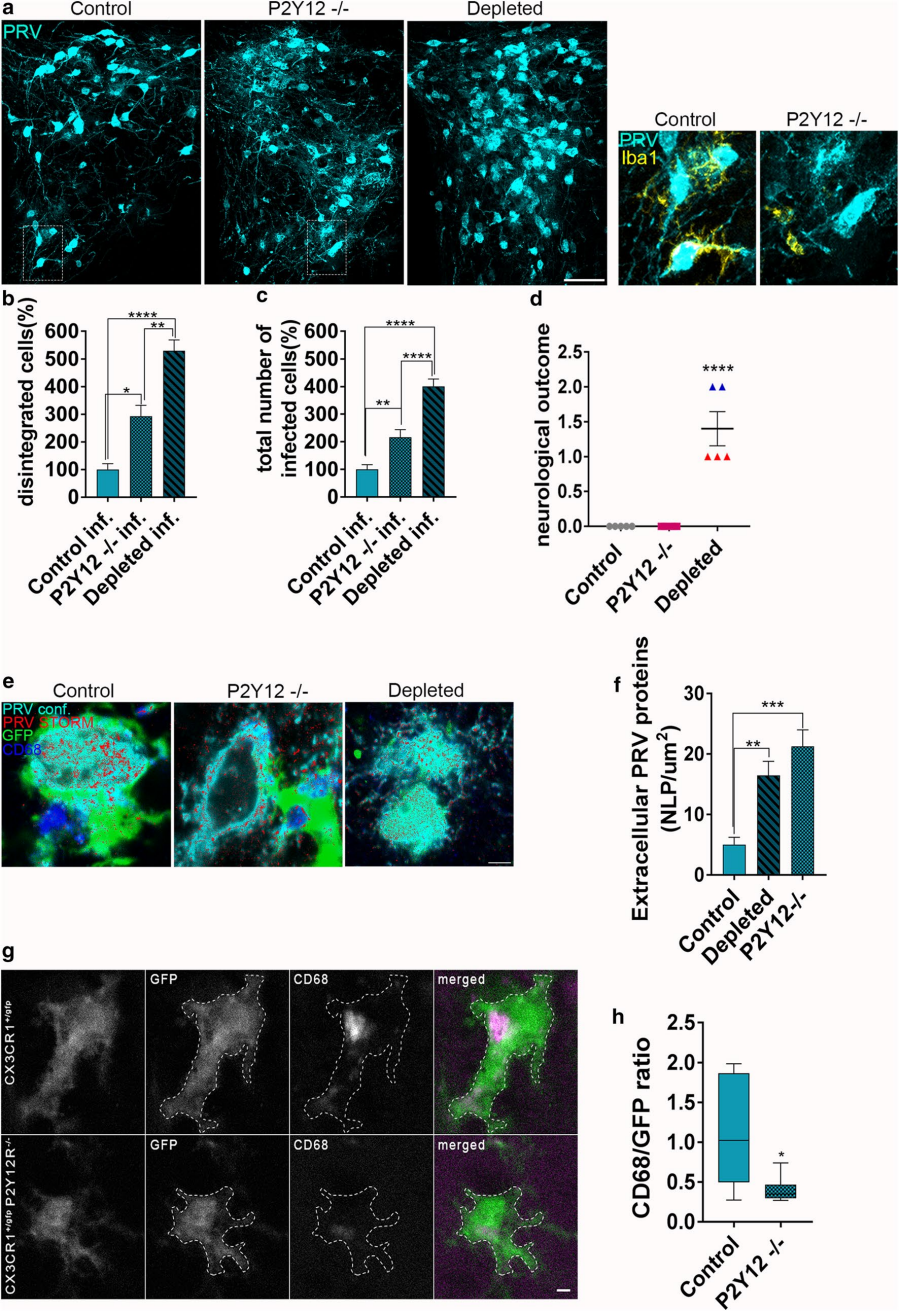
The absence of P2Y12-positive microglia leads to increased neuronal infection, impaired phagocytosis and the accumulation of extracellular virus particles. **a** PRV immunofluorescence showing a marked increase in the number of infected (PRV-positive) neurons in P2Y12^−/−^ mice and after microglia depletion, compared to control animals. Note the lack of recruited Iba1 + microglia (yellow) to PRV-positive neurons in P2Y12^−/−^ mice (inserts). **b** Numbers of infected (PRV +), disintegrated neurons in control, P2Y12^−/−^ and microglia-depleted mice. **c** Total number of infected (PRV +) neurons is significantly higher in P2Y12^−/−^ and microglia-depleted mice. **d** An absence of microglia, but not P2Y12-deficiency results in rapidly deteriorating neurological symptoms 5 days after PRV infection (0: no symptoms, 1: drooling and heavy breathing, 2: seizures and muscle spasms). **e** STORM super-resolution microscopy reveals a marked increase in extracellular PRV proteins in both P2Y12^−/−^ and microglia-depleted mice compared to wild type mice. **f** Quantitative assessment of extracellular PRV proteins on STORM images. Number of extracellular PRV-positive localisation points (NLP) is shown in each group. **g** Images showing microglial phagolysosomes identified by CD68 immunofluorescence in control Cx3Cr1^+/gfp^, and P2Y12-deficient (Cx3Cr1^+/gfp^ x P2Y12^−/−^) mice. **h** P2Y12-deficient microglia show a significant reduction of CD68-positive phagolysosomes compared to wild-type microglia (CD68 immunofluorescent integrated density/GFP immunofluorescent integrated density within microglial cell bodies, *p* = 0.0322, Mann–Whitney *U* test, *n* = 35 cells/16 ROIs). Data on d expressed as median and interquartile range, otherwise as mean ± s.e.m. **b**, **c**, **d** Mann–Whitney *U* test, *n* = 5–6. Scale bars: **a** 100 µm; **e** and **g** 2 µm. All data expressed as mean ± s.e.m **b** Control vs P2Y12 *p < 0.05; Control vs Depleted ****p < 0.0001; P2Y12 vs Depleted **p < 0.001, One-Way ANOVA **c** Control vs P2Y12 **p < 0.001; Control vs Depleted ****p < 0.0001; P2Y12 vs Depleted ****p < 0.0001 One-Way ANOVA **d** Control vs Depleted ****p < 0.0001; P2Y12 vs Depleted ****p < 0.0001 One-Way ANOVA **f** Control vs Depleted **p <  0.001; Control vs P2Y12 ***p < 0.0001 One-Way ANOVA

Despite the markedly increased number of infected neurons in P2Y12^−/−^ mice, no neurological symptoms have been observed (Fig. 5i), suggesting that the absence of microglia (Fig. 1q), but not of microglial P2Y12 alone, can cause the adverse neurological outcome in this model. To confirm this and to test for possible mechanisms underlying this difference, a new study was performed enabling a direct comparison of control, P2Y12^−/−^ and microglia-depleted mice after infection. In P2Y12^−/−^ mice there was deficient recruitment of microglia to infected neurons. As earlier, both the absence of P2Y12 and microglia depletion caused marked elevations in the numbers of infected and disintegrated neurons, but both these measures were elevated significantly more in the microglia-depleted animals (Fig. 6a–c). Histological analysis on cresyl violet-stained brain sections also demonstrated significantly increased neuronal injury/loss in both P2Y12^−/−^ and microglia-depleted animals with highest levels seen after microglia depletion [Suppl Fig. 8 (Online Resource 1)]. However, the neurological symptoms only emerged with microglia depletion (Fig. 6d). In contrast, levels of extracellular virus proteins were identical in P2Y12^−/−^ and microglia-depleted mice, while markedly increased compared to control mice (Fig. 6e, f). P2Y12^−/−^ microglia did show significantly lower levels of CD68-positive phagolysosomes compared to that seen in control animals (Fig. 6g, h), indicating the lack of normal phagocytic activity in the absence of P2Y12.

### Microglia recruit leukocytes into the brain in response to virus infection independently of P2Y12-mediated signalling

**Fig. 7:**
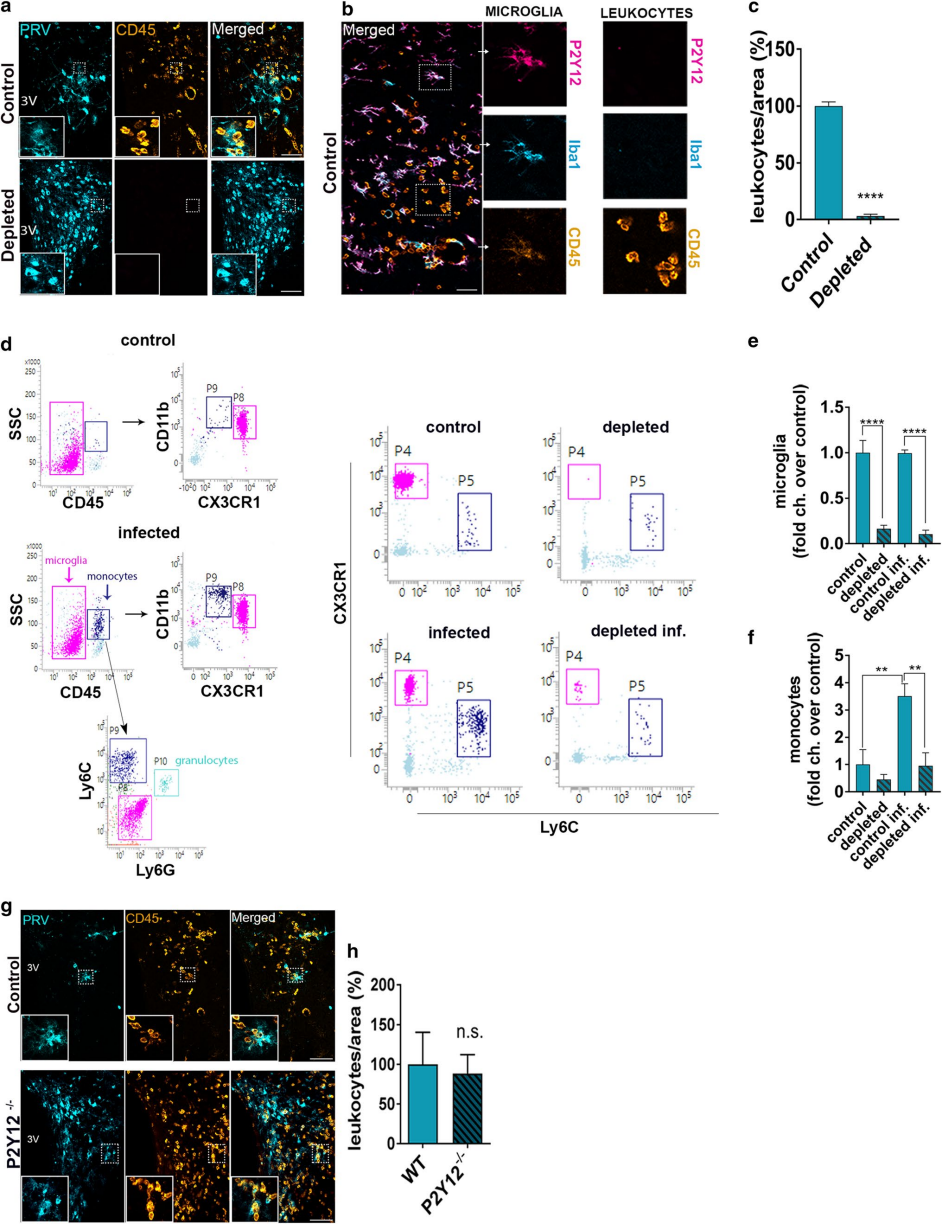
Microglia are instrumental for leukocyte recruitment into the brain in response to virus infection, which is independent of P2Y12-mediated signaling. **a** Recruitment of CD45-positive leukocytes (orange) to the hypothalamic paraventricular nucleus (PVN) is seen around virus-infected neurons (cyan), which is markedly reduced by selective elimination of microglia **b** P2Y12 expression (magenta) discriminates microglia from blood-borne cells expressing high levels of CD45 (orange), most of which are negative for the microglia/macrophage marker Iba1 (cyan). **c** Leukocyte numbers are significantly lower in microglia depleted animals, area: 0,2 mm^2^, **d** Flow cytometric dot plots showing that microglia (Cx3Cr1^high^, CD45^low^ Lyc6^−^ cells, P8) can be well characterized and separated from infiltrating monocytes (Cx3Cr1^+/int^, CD45^high^, Ly6C^high^, Ly6G^−^ cells, P9), during viral infection in the brain. The almost complete absence of microglia is seen after selective microglia depletion (gates P4). In spite of the exaggerated virus infection in the absence of microglia, CD45^high^ blood-borne leukocytes (P9 gate), specifically monocytes (CD45^high^, Cx3Cr1^+^, CD11b^+^, Lyc6^high^ cells, P5 gate) are profoundly reduced in the microglia depleted brains. **e** Cx3Cr1^+/gfp^ microglia are profoundly reduced in the brain after feeding mice the CSF1R inhibitor PLX5622 for 3 weeks. **f** Monocyte numbers increase significantly in response to virus infection in the brain, which is markedly reduced in microglia depleted animals. **g** Immunofluorescence shows infiltrating CD45-positive leukocytes (orange) around virus-infected neurons (cyan) in the paraventricular nucleus. **h** Numbers of infiltrating leukocytes (orange) in P2Y12^−/−^ mice are not significantly different from control animals, area: 0,2 mm^2^ All data expressed as mean ± s.e.m **c,** ****p < 0.0001 unpaired *t* test *n* = 12; **e** control vs depleted ****p < 0.0001, control inf. vs depleted inf. ****p < 0.0001 One-Way ANOVA; **f** control vs control inf. **p < 0.001, control inf. vs depleted inf. **p < 0.001 One-Way ANOVA. **h** n.s. not significant Scale bar **a**, **b**, **g**, 50 µm

Since previous studies have shown that blood-borne cells are recruited to the brain after virus infection [15, 48], we wondered whether microglia and P2Y12-mediated actions are involved in neuroinflammatory and neurobehavioral changes in this model. As expected, numerous round-shaped or elongated leukocytes with high CD45 immunopositivity were recruited to sites of virus infection (Fig. 7a). These were clearly discriminated from microglia based on their higher CD45 expression, morphology, the absence of the microglia/macrophage marker Iba1 from the majority of the cells and the complete absence of P2Y12, which is a microglia-specific marker in the brain [Fig. 7b, Suppl Fig. 7 (Online Resource 1)]. P2Y12 is known to be expressed at high levels by microglia compared to monocytes or monocyte-derived macrophages [45]. Surprisingly, selective elimination of microglia resulted in a profound reduction in CD45-positive leukocytes at sites of virus infection (Fig. 7a, c), despite the increased number of infected neurons compared to that seen in control mice (Fig. 1g). This was not due to changes in peripheral leukocyte populations in response to PLX5622, since elimination of microglia by PLX5622 did not cause a significant reduction in circulating or splenic myeloid cell populations including monocytes, granulocytes and macrophages, and did not affect numbers of T cells and B cells [Suppl. Figs. 9 and 10 (Online Resource 1)], confirming our earlier results obtained by another CSF1R antagonist, PLX3397 [56]. Infiltrating leukocyte populations have also been characterized by flow cytometry. The main population of CD45-positive cells recruited in response to virus infection were monocytes (CD45^high^, Cx3Cr1^+^, CD11b^+^, Ly6C^high^, Ly6G^−^ cells), which population was markedly reduced in the absence of functional microglia (Fig. 7d–g). A non-significant trend to increased CD8 T cells in the brain in response to infection was also observed, which was not influenced by microglia depletion [Suppl. Fig. 11 (Online Resource 1)]. We also found that microglia exposed to PRV in vitro produced (CCL5) RANTES and MCP-1, whereas CCL5 and IL-1α were significantly reduced in hypothalamus homogenates of infected mice after microglia depletion [Suppl. Fig. 13 (Online Resource 1)]. Importantly, exaggerated virus infection in the brain was associated with an increase in circulating granulocytes in microglia depleted mice, suggesting that peripheral myeloid populations were capable of responding to virus infection, but their recruitment into the brain was inhibited by the absence of microglia [Suppl. Fig. 9 (Online Resource 1)]. Next, we investigated whether purinergic signalling through P2Y12 in microglia could contribute to leukocyte recruitment into the brain in response to virus infection. Importantly, no changes in the numbers of CD45-positive, blood-borne leukocytes were seen in P2Y12^−/−^ mice after infection and monocyte infiltration was not impaired [Fig. 7g, h; Suppl. Fig. 12a, c, (Online Resource 1)]. Thus, microglia appear to be key inducers of monocyte recruitment into the brain, but these processes are largely independent of microglial P2Y12-mediated mechanisms that play a key role in controlling the spread of virus infection.

### Recruitment of P2Y12-positive microglia and leukocytes at sites of infection in the human brain during herpes simplex encephalitis

**Fig. 8:**
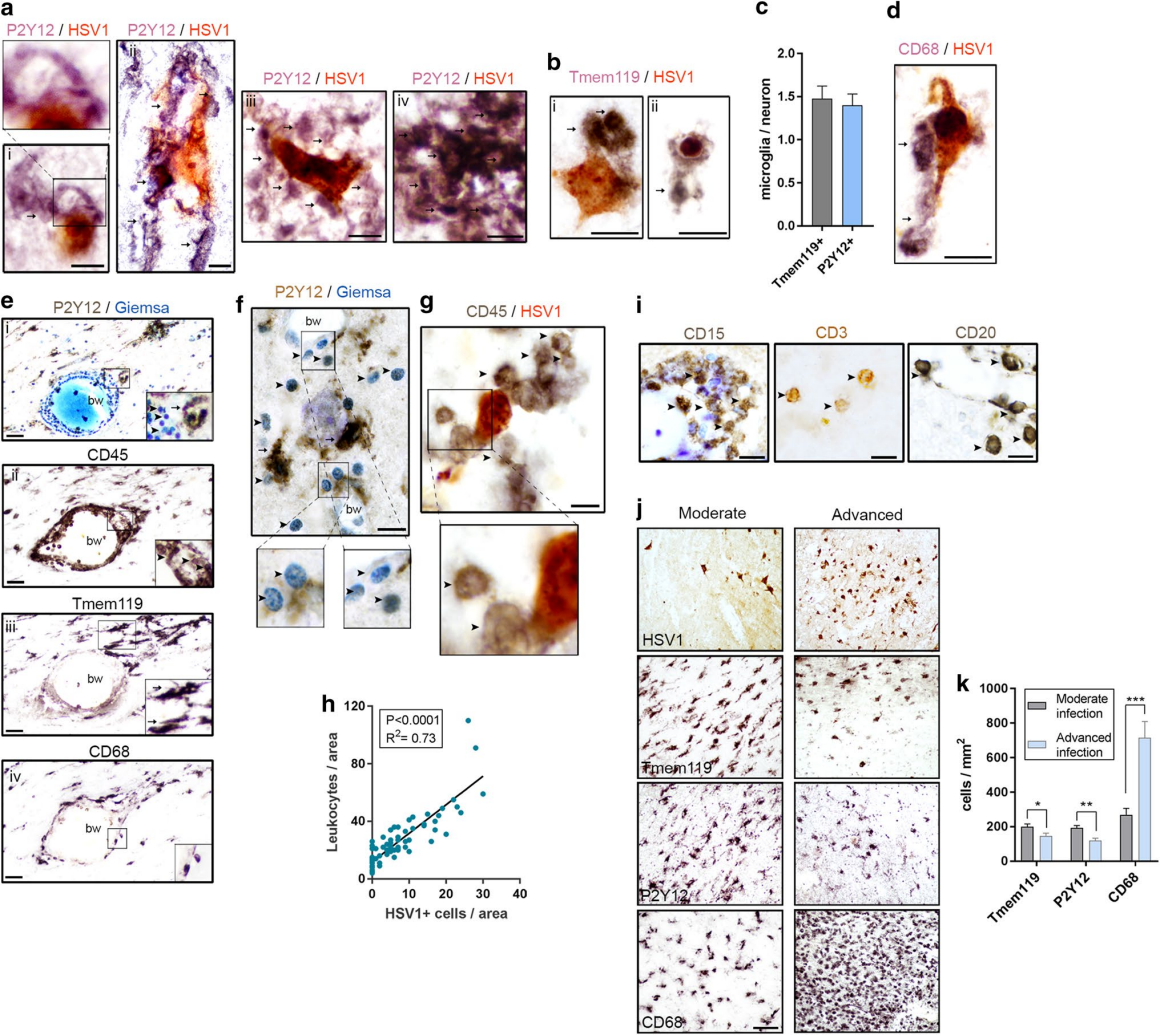
Microglia and leukocytes are recruited to infected neurons in the human brain. **a** Different stages of recruitment of P2Y12 + microglia (arrows, visualized by DAB-Ni) are observed around herpes simplex-1 (HSV1)-infected cells (brown, visualized by NovaRED HRP substrate) in the human cerebral cortex. Processes of P2Y12-positive microglia contact HSV-positive neurons (i–ii) and assemblies of microglial cells are seen around infected cells (iii). Groups of P2Y12-positive microglia displaying amoeboid, phagocytic morphology in the absence of detectable HSV antigens are also found in infected areas (iv). **b**. Microglia labelled with Tmem119 (arrows) are recruited to HSV1-positive neurons (i) and engulf HSV-positive cells (ii). **c** Quantification showing the average number of Tmem119-positive or P2Y12-positive microglia contacting HSV1-positive cells. **d** CD68-positive brain macrophages (DAB-Ni) surround infected neurons (NovaRED). **e** Viral infection is associated with the recruitment of leukocytes (arrowheads) visualized by Giemsa stain (i) and CD45 immunostaining (ii) in the vicinity of microglia (arrows) labelled with P2Y12 (i) or Tmem119 (iii). CD68-positive macrophages in close association with blood vessels and parenchymal CD68-positive cells with microglial morphology suggest a mixed (blood-borne and resident) origin of brain phagocytes (iv). **f** In areas of virus infection, neurons surrounded by P2Y12-positive microglia are associated with leukocytes (Giemsa, arrowhead) and **g** CD45-positive leukocytes are recruited to HSV1-positive cells. **h** Leukocyte numbers in infected brain areas show correlation with the number of virus-infected cells. *p* < 0.0001, linear regression, *n* = 64 FOV, from 5 patients. **i** CD15-positive myeloid cells, and in lower amount, CD3-positive lymphocytes and CD20-positive B cells are observed at areas of HSV1 infection. **j** Moderate HSV1 infection (less than 50 HSV + cells/mm^2^) is associated with the presence of numerous Tmem119-positive, P2Y12-positive microglia, and CD68-positive microglia/macrophages, whilst decreased numbers of Tmem119 and P2Y12-positive cells. In advanced HSV1 infection (50–500 HSV + cells/mm^2^), markedly increased numbers of CD68-positive brain macrophages are observed in the brain parenchyma. **k** Quantification of parenchymal microglia and CD68-positive brain macrophages in the case of moderate and advanced HSV1 infection. bw—blood vessel. Scale bars: **a**, **b**, **d**, **f**, **g**, **h**, **i**—10 μm; **e** and **j**—50 μm. **k** *p < 0.05, **p < 0.001, ***p < 0.0001

To investigate microglia recruitment and neuroinflammatory changes in the human brain, we analysed herpes simplex type 1 (HSV-1) encephalitis temporal lobe samples in which infection had been confirmed both by PCR and immunohistochemistry as reported earlier [13]. Processes of P2Y12-positive human microglial cells were extended to HSV-1-positive cells, and infected neurons were surrounded by activated microglial cells [Fig. 8a, Suppl. Table 1 (Online Resource 1)]. This has been confirmed with another specific microglial marker, Tmem119 (Fig. 8b). Infected cells were contacted by 1–3 microglia (on average 1.5 microglia/HSV1 + cell, Fig. 8c). Groups of recruited, amoeboid cells showing CD68-immunopositivity indicating phagocytic activity were also observed at sites of virus infection (Fig. 8d). Microglial cells were negative to HSV antigens suggesting that productive virus infection does not develop in these cells. Leukocytes identified by CD45-immunohistochemistry and Giemsa staining, whilst being P2Y12- and Tmem119-negative have been found at sites of virus infection in close vicinity of HSV-1-positive cells and microglia (Fig. 8e–g). A strong positive correlation between leukocyte numbers and HSV-1-positive cells was also observed (Fig. 8h). Populations of CD15-positive myeloid cells, and in lower amount, scattered CD3-positive lymphocytes and CD20-positive B cells were identified at areas of HSV1 infection (Fig. 8i). In the absence of HSV1 infection, the vast majority of Iba1-positive microglia was found to be P2Y12 positive (96%) and numbers of Tmem119-positive and P2Y12-positive microglia were similar in the brain parenchyma [Suppl. Fig. 14 (Online Resource 1)]. We found that moderate HSV1 infection (less than 50 HSV1-positive cells/mm^2^) was mostly associated with the activation of local microglia which were Tmem119- and P2Y12-positive. CD68-positive cells with either ramified or amoeboid morphology were also observed in these areas. At areas of advanced HSV1 infection (50–500 HSV1-positive cells/mm^2^), numerous CD45-positive cells were observed in the brain parenchyma, which was associated with markedly increased numbers of CD68-positive-macrophages (likely to be of both microglial and blood-borne origin). In line with this, the number of ramified microglia, and the total number of P2Y12-positive or Tmem119-positive cells was reduced [Fig. 8j, k and Suppl. Fig. 15. (Online Resource 1)]. Interestingly, similar reduction in microglial numbers was seen in mice at areas showing heavy virus load at the advanced stages of virus infection in the brain [Suppl. Fig. 16 (Online resource 1)].

## Discussion

Here, we show that microglial P2Y12-mediated responses are essential for the recognition and effective elimination of compromised neurons after virus infection that reaches the brain exclusively via retrograde transsynaptic spread. Microglia recruitment occurs within a few hours in vivo and leads to the phagocytosis of infected cells. Marked increases in the number of disintegrated cells and leakage of viral antigens into the extracellular space are seen in the absence of functional microglia, which are associated with exaggerated infection and the development of neurological symptoms. We also show that P2Y12 receptors are key drivers of microglial phagocytosis both in vivo and in vitro and that microglial P2Y12 is essential for appropriate responses to nucleotides released by infected neurons in the brain. Furthermore, we identify microglia as key inducers of monocyte recruitment into the brain in response to neurotropic virus infection and also demonstrate the relevance of these findings in the human brain.

Signals mediating early recognition of injured cells and those inducing phagocytosis of synapses or neurons by microglia in the brain are poorly defined, and the mechanisms of microglial decision-making regarding the fate of injured neurons are presently unclear. Microglia may sense changes in neuronal activity via altered extracellular ion gradients, CX3CL1-CX3CR1 or CD200-CD200R interactions, purinergic signalling, pannexin-1 hemichannels and other mechanisms to shape synaptic connectivity and neuronal networks under both physiological or pathological conditions [3, 28, 36, 53]. In addition, we and others have shown that ’danger signals’ from injured cells in the brain such as DNA, HMGB1, heat shock proteins or ATP act as potent activators of microglia and also contribute to the outcome in different brain pathologies [16, 21, 34, 35]. However, due to the complexity of these signalling pathways and the immediate response of microglia to any tissue disturbance, it is difficult to dissect the mechanisms through which microglia recognize stressed neurons in most experimental models of brain injury.

To establish a model of neuronal injury in which microglial responses to local signals can be studied within a realistic time frame and without in situ manipulation of the brain microenvironment, we have used genetically modified PRV-Bartha derivatives, which exhibit precisely controlled, retrograde transneuronal spread but do not infect microglia [6, 50]. In addition, these recombinant PRV strains allowed precise time-mapping of the spread of infection due to the expression of reporter proteins with different kinetics [4, 6]. At the same time, we could investigate the functional contribution of microglia to neurotropic herpesvirus infection, which has not been previously investigated using selective elimination of microglia. Since immune surveillance by circulating immune cells is restricted in the brain parenchyma [52], early recognition of infection by microglia is likely to be critical to mount an appropriate immune response. The central inflammatory response induced by neurotropic herpesviruses including microglial activation and recruitment of blood-borne immune cells has been previously characterized by excellent earlier studies [19, 48, 50] and our former data has also shown that microglia surround infected neurons in the brain [15]. Recent reports highlighted the importance of central type I interferon responses against vesicular stomatitis virus and herpes simplex virus type 1 implicating microglia as a source of inflammatory mediators in anti-viral immunity in the brain [17, 49]. However, the kinetics and the mechanisms of microglia recruitment to infected cells have remained unexplored to date, similarly to the need for phagocytic activity by microglia to control the spread of infection. Our data obtained both in vivo in real time and in vitro shows that the rapid and precisely controlled migration of functional microglia is critical not only to limit the spread of infection in the brain, but timely elimination of infected neurons is essential to prevent contact infection and to control the leakage of viral particles and antigens into the brain parenchyma. The rapidly worsening neurological symptoms of mice in the absence of microglia, but not in P2Y12-deficient mice, may be due to both exaggerated infection and the lack of microglial factors that control neuronal activity in the injured brain [3, 38, 56], which should be investigated in further studies. Since the PRV Bartha-Dup strains show highly specific neurotropism in vivo [4–6] and we did not find any sign of hematogenous dissemination of infection or immunopositivity to viral antigens in the liver or the spleen even after PLX5622 treatment, a major role of peripheral immune mechanisms in the markedly increased spread of infection in microglia-depleted mice is unlikely.

Infected cells, including neurons and microglia were reported to sense HSV-1 via cytoplasmic DNA sensors, namely the adaptor protein stimulator of type I IFN genes (STING) [49]. However, the signals initiating microglia recruitment to infected neurons in the absence of microglial infection had remained unclear. Our in vivo and in vitro data suggest that soon after the development of productive infection, purinergic mediators released from neurons recruit the processes of uninfected microglia in their vicinity, followed by the displacement of the cells, leading to the formation of tight membrane–membrane interactions with the infected neurons. Since PRV infection alters neuronal activity [42], we hypothesised that the earliest signals from infected neurons to microglia are more likely to include mediators regulating rapid microglia–neuron interactions in vivo than de novo production of inflammatory chemokines. Specifically, noxious stimuli in neurons can trigger a sustained increase of extracellular ATP, which results in microglial activation and recruitment within minutes to hours [14, 22]. In fact, our data shows that purine nucleotides released from affected neurons contribute to microglial process extension, cell migration to infected neurons and subsequent phagocytic activity via microglial P2Y12 receptors. ATP released from injured cells leads to the activation of P2-type and adenosine receptors upon extracellular ATP catabolism by ecto-nucleotidases [51]. In line with this, we observed increased ecto-ATPase levels and NTDPase1 activity in infected cells and microglia. ATP is a strong chemotactic signal for microglia in vivo [14] and hydrolysis of ATP to ADP, which is the main ligand for P2Y12 takes place by ecto-nucleotidases within minutes [51, 55]. In turn, increased P2Y12 receptor levels were found on microglial processes contacting infected neurons, as assessed by super-resolution microscopy. Since infected neurons at the stage of immediate-early reporter protein expression are viable and electrophysiologically active [42], these results also imply that microglia are well-equipped to identify injured neurons way earlier than the integrity of the cell membranes is compromised. Our ultrastructural analysis and in vitro data also confirm this, showing normal cell membrane integrity until late stages of virus infection. Thus, in spite that P2Y12 has been implicated earlier in the recruitment of microglia to sites of tissue injury in the brain [14, 25], the present in vivo and in vitro studies have identified the cell-autonomous effect of P2Y12 on microglia to rapidly recognize and eliminate infected neurons for the first time. We also show that P2X7, which plays a major role in microglial inflammatory responses and cytokine production [54] is dispensable for anti-viral immunity in this experimental model.

We also identify microglia as key contributors to monocyte recruitment to the brain during virus infection. Previous studies have implicated activated microglia in leukocyte recruitment into the brain upon virus infection, and showed that antibodies to CXCL10 and CCL2 (MCP-1) reduce the migration of murine splenocytes toward HSV-infected microglia in vitro [40, 41]. In our experimental model, elimination of microglia by CSF1R blockade was highly selective, as it did not have a significant impact on circulating and splenic leukocytes (including myeloid cell types), and infection-induced increases in circulating granulocytes was preserved in PLX5622-treated mice. In contrast, recruitment of monocytes to the brain was almost completely abolished in microglia depleted mice. In these studies, we made use of both CD45 and Cx3Cr1 as markers to reliably discriminate microglia (CD45^low^, Cx3Cr1^high^, Ly6c^−^ cells) from monocytes (CD45^high^, Cx3Cr1^+^, Ly6c^high^ cells) without the need of complex BM chimeric studies that inherently include changes in BBB function and may cause microglia activation [62]. Importantly, microglial P2Y12 was essential to mediate microglia recruitment and phagocytosis, but was dispensable for monocyte recruitment to the brain. These data suggest that other microglial chemotactic factors (such as MCP-1 or RANTES) could be responsible for driving leukocyte migration to sites of infection and injury in the brain, which should be investigated in further studies. Since monocyte recruitment in P2Y12^−/−^ mice was identical to that seen in control animals, but both an absence of microglia and P2Y12 deficiency resulted in markedly enhanced spread of infection, blood-borne monocytes may not significantly limit viral spread in the current experimental model. A similar conclusion was presented in a model of corona virus infection induced by direct injection of the virus into the brain, in spite that reduction of microglia numbers was associated with higher number of blood-borne macrophages in this study [61]. Histological characterization of post-mortem samples from human HSV-1 encephalitis cases also suggests that P2Y12-positive microglia isolate infected neurons, and a marked increase in leukocyte recruitment was also associated with severe infection.

Microglia depletion, but not P2Y12 deficiency leads to characteristic neurological symptoms in virus-infected mice. In line with this, microglia-depleted mice had higher numbers of infected/dying neurons than that seen in P2Y12^−/−^ mice, while the levels of extracellular virus proteins were not different, although significantly increased in both groups compared to control mice. Thus, the rapidly deteriorating neurological outcome seen in microglia-depleted animals may be partially due to the markedly increased neuronal infection and to the absence of potentially neuroprotective microglial mediators, such as interleukin-10 [23]. While our data show that P2Y12-dependent mechanisms are instrumental to limit neurotropic virus infection in the brain, additional microglial receptors could also contribute to this process. Since P2Y12^−/−^ mice showed comparable leukocyte infiltration to control animals, while microglia depletion markedly influenced leukocyte responses, a role for blood-borne cells in shaping neurobehavioral symptoms seen in this experimental model cannot be fully excluded.

**Fig. 9:**
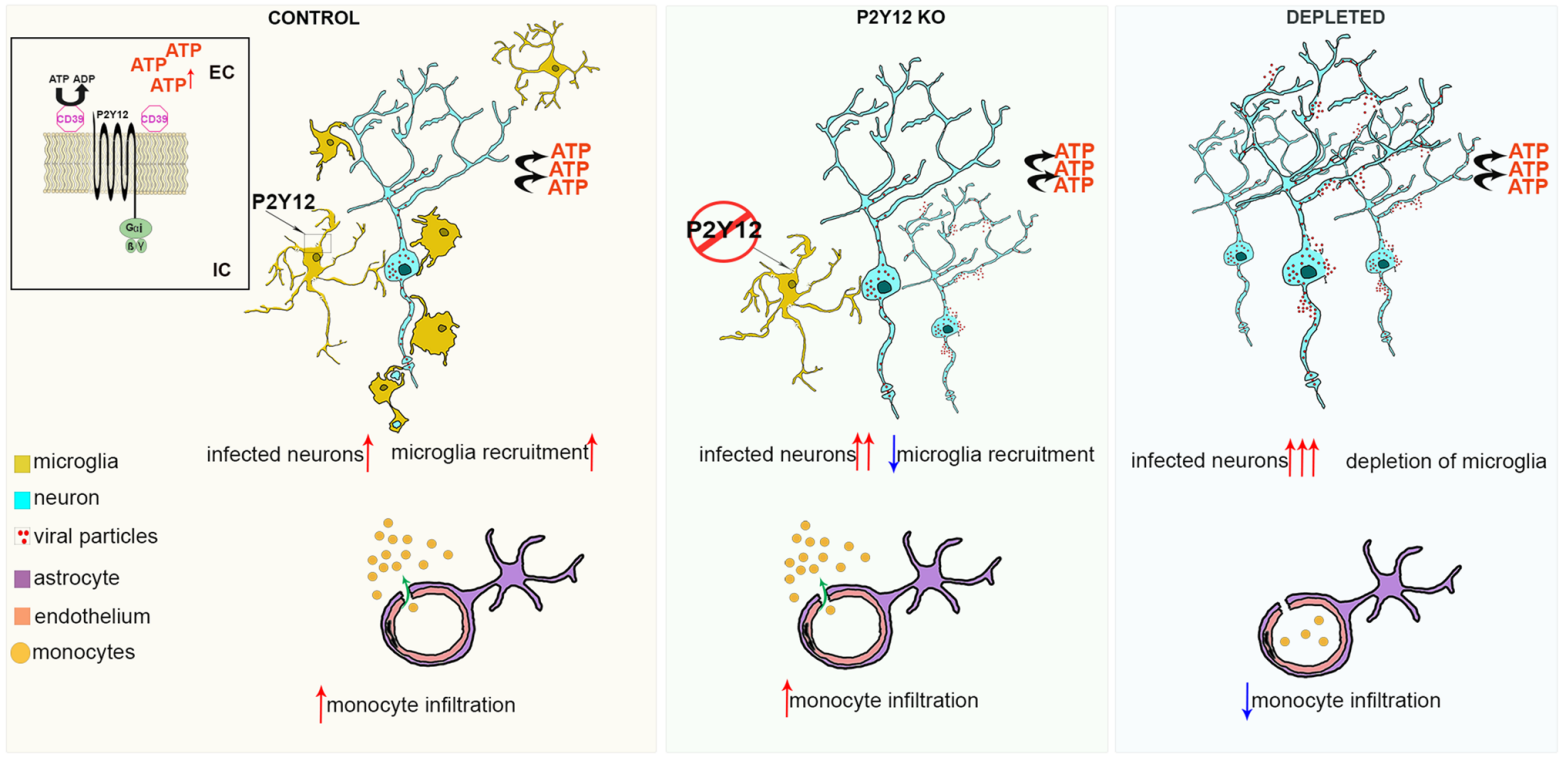
Summary of neuroinflammatory changes after virus infection. In microglia-competent control mice, microglia recruitment around BDG-infected neurons is observed, which is associated with monocyte recruitment at sites of infection. ATP released by compromised neurons may be cleaved by CD39 to ADP leading to stimulation of P2Y12 receptors and act as a trigger for microglia recruitment and phagocytosis. The lack of microglia leads to markedly increased numbers of viral infected neurons and an almost complete absence of monocyte recruitment. The lack of microglial P2Y12 leads to reduced microglial recruitment compared to control animals, suggesting the pivotal role of P2Y12 receptor in this process, whilst monocyte infiltration is comparable to that seen in control animals

The implications of these data for neurological diseases are far-reaching. The findings that microglia control neurotropic virus infection via P2Y12 in mice and the recruitment of P2Y12-positive microglia to HSV-1 cells was observed in the human brain suggest that microglial P2Y12 could play in general an important role in anti-viral immunity in the CNS (Fig. 9). Beyond infectious diseases caused by alphaherpesviruses such as PRV in swine or HSV-1 in humans, other viruses such as rabies, Zika virus, Alphaviruses, West-Nile virus, Epstein Barr virus, Influenza A viruses, and Enteroviruses can exhibit neurotropism and cause diverse neuropathologies in both humans and rodents [33, 38, 60]. In addition, the emerging role of neurotropic viruses in many forms of neurodegeneration [33, 38, 64] and the common molecular fingerprints of cellular injury suggest that understanding the mechanisms through which microglia control the elimination of injured neurons in the brain could facilitate the development of targeted therapies in several common brain diseases.

## Electronic supplementary material

Below is the link to the electronic supplementary material.

Supplementary material 1 (PDF 3084 kb)

Supplementary material 2 (AVI 4945 kb)

Supplementary material 3 (AVI 8378 kb)

Supplementary material 4 (AVI 1598 kb)

Supplementary material 5 (AVI 2780 kb)

Supplementary material 6 (AVI 7530 kb)

Supplementary material 7 (AVI 8165 kb)

Supplementary material 8 (AVI 9677 kb)

Supplementary material 9 (AVI 1040 kb)

Supplementary material 10 (AVI 1241 kb)

Supplementary material 11 (AVI 2483 kb)

Supplementary material 12 (AVI 2362 kb)
