# Supplementary material for: Microglia control the spread of neurotropic virus infection via P2Y12 signalling and recruit monocytes through P2Y12-independent mechanisms
Source: Acta Neuropathol. 2018 Jul 19;136(3):461–82. doi: 10.1007/s00401-018-1885-0 (PMC6096730; doi:10.1007/s00401-018-1885-0)
Supplement: Supplementary file 1 — Supplementary material 1 (PDF 3084 kb) [file 401_2018_1885_MOESM1_ESM.pdf]

Online Resource Supplemental Material 1

Acta Neuropathologica

Microglia control the spread of neurotropic virus infection via P2Y12 signalling and recruit monocytes through P2Y12-independent mechanisms

Rebeka Fekete[1], Csaba Cserép[1], Nikolett Lénárt[1], Krisztina Tóth[1], Barbara Orsolits[1], Bernadett Martinecz[1], Előd Méhes[2], Bálint Szabó[2], Valéria Németh[2], Balázs Gönci[2], Beáta Sperlágh[3], Zsolt Boldogkői[4], Ágnes Kittel[3], Mária Baranyi[3], Szilamér Ferenczi[5], Krisztina Kovács[5], Gergely Szalay[6], Balázs Rózsa[6], Connor Webb[1], Gabor G. Kovacs[7], Tibor Hortobágyi[8], Brian L. West[9], Zsuzsanna Környei[1] and Ádám Dénes[1,*]

1. “Momentum” Laboratory of Neuroimmunology, Institute of Experimental Medicine, Hungarian Academy of Sciences, Budapest, Hungary
2. Department of Biological Physics, Eötvös University, Budapest, Hungary
3. Laboratory of Molecular Pharmacology, Institute of Experimental Medicine, Hungarian Academy of Sciences, Budapest, Hungary
4. Department of Medical Biology, Faculty of Medicine, University of Szeged, Szeged, Hungary
5. Laboratory of Molecular Neuroendocrinology, Institute of Experimental Medicine, Hungarian Academy of Sciences, Budapest, Hungary
6. Laboratory of 3D Functional Network and Dendritic Imaging, Institute of Experimental Medicine, Hungarian Academy of Sciences, Szigony u. 43., 1083 Budapest, Hungary
7. Institute of Neurology, Medical University of Vienna, Vienna, Austria and Neuropathology and Prion Disease Reference Center, Semmelweis University, Budapest, Hungary
8. MTA-DE Cerebrovascular and Neurodegenerative Research Group, University of Debrecen, Debrecen, Hungary & Institute of Psychiatry Psychology and Neuroscience, King’s College London, London, UK
9. Plexxikon Inc., Berkeley, CA 94710, USA

***Correspondence:** Dr. Ádám Dénes, Institute of Experimental Medicine, Szigony u. 43, 1083 Budapest, Hungary. Email: denes.adam@koki.mta.hu; Phone: +36 209549149

This PDF file includes:

- Supplementary Figures 1-16
- Supplementary Table 1
- Legends for Supplementary Videos 1-11
- Supplementary Methods

Other Supplementary Materials for this manuscript includes the following:

- Supplementary Videos 1-11

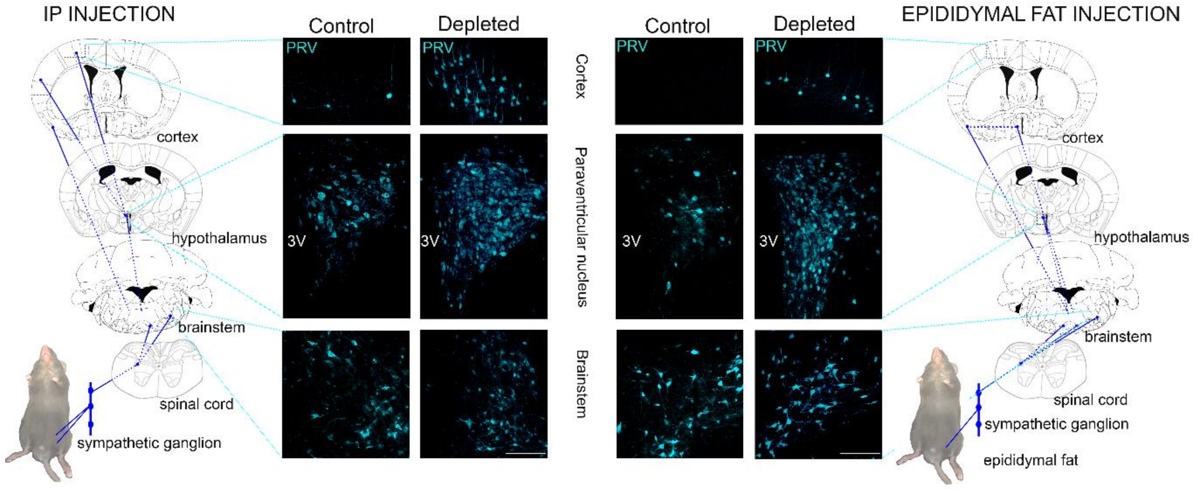

Supplementary Figure 1. The impact of microglia depletion on the spread of virus infection in the brain. Bartha-DupGreen (BDG) was injected intraperitoneally or into the epididymal white adipose tissue (left side) to initiate retrograde transsynaptic spread of infection into the CNS, with or without the selective depletion of microglia with PLX5622. The propagation of infection in the brain was assessed 5 days after infection. Note the marked increase of PRV-immunopositive neurons (cyan) in the brain in the absence of microglia. This includes the spread of infection to higher cortical areas, including the piriform cortex, the insular cortex and the primary motor cortex after intraperitoneal injection. Due to the primarily sympathetic autonomic innervation of the white adipose tissue, the infection affects mostly the piriform cortex in microglia depleted animals, whereas the virus does not the reach the cerebral cortex in control mice. 3V – third ventricle. Maximum Intensity Projection from confocal Z stack of 5 steps were made with 3,38 µm (cortex), 0,83 µm (PVN), 4,05 µm (brainstem) step size. Scale bars: 50 µm.

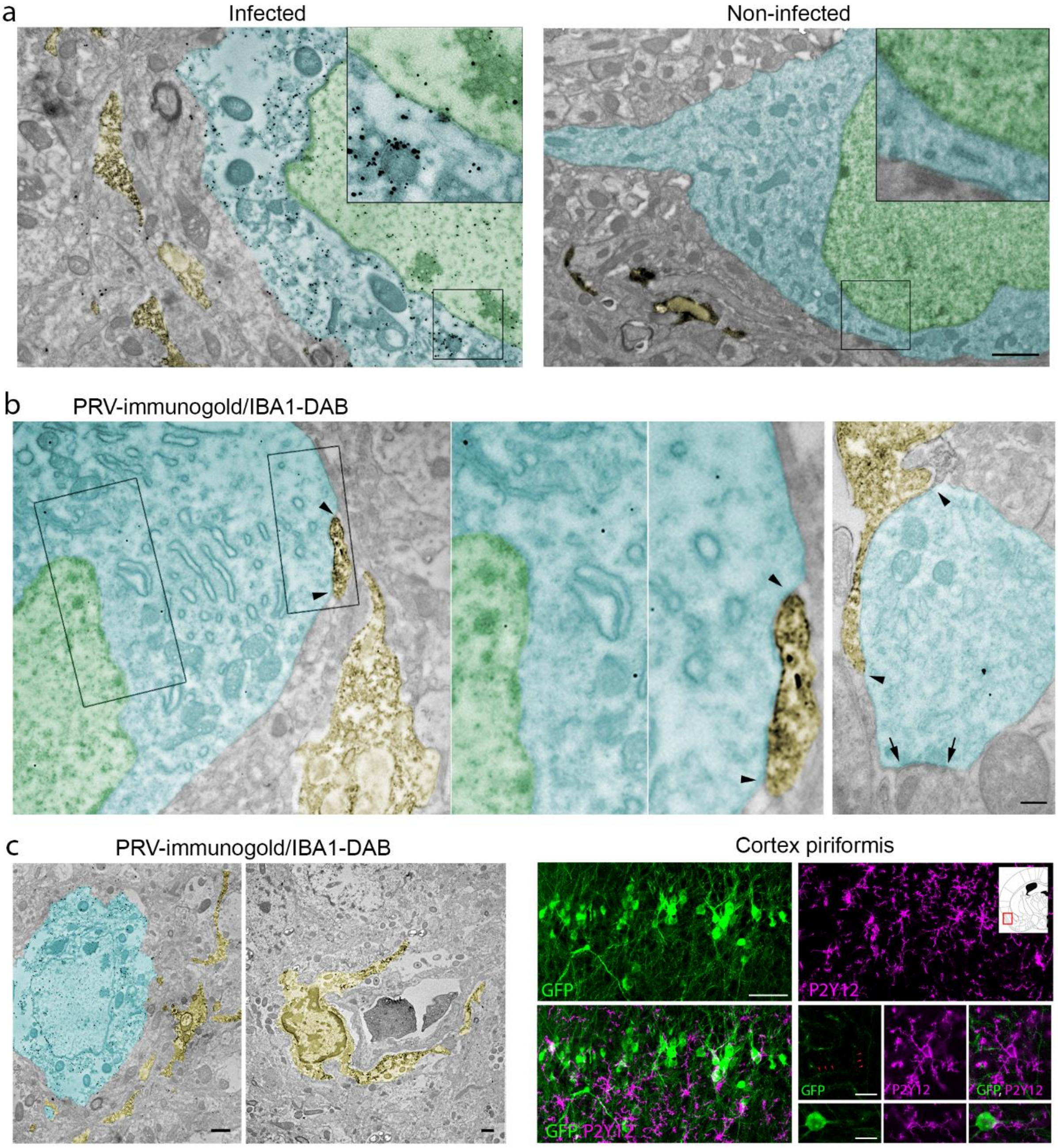

Supplementary Figure 2. Pseudorabies virus infects neurons but does not induce productive infection in microglial cells *in vivo*. a) PRV-immunolabeling is completely absent in virus-free animals. Transmission electron microscopic (TEM) images showing the presence of anti-PRV immunogold labeling in infected, and its complete absence in non-infected animals. Microglial processes (dark DAB-precipitate, yellow pseudocolor) are found in the vicinity of neurons (cytoplasm: cyan pseudocolor, nucleus: green psudocolor). The infected neuron contains a high level of anti-PRV immunogold particles (black grains), viral capsids and mature virions (insert). The non-infected animal is void of viral particles and PRV-immunogold particles, confirming the specificity of the antibody. Scale bar: 1 µm. b) TEM images show that microglial processes (dark DAB-precipitate, yellow pseudocolor) find and contact PRV-infected neurons even at the early phases of infection, when only low levels of anti-PRV immunogold labeling is present (cytoplasm: cyan pseudocolor, nucleus: green psudocolor). Arrowheads mark sites of microglial contacts, arrows point to a synapse on the

dendrite of an infected neuron. Scale bar: 300 nm on left panel, 200 nm on right panel. c) TEM pictures (first panel) show that DAB-positive microglia (yellow pseudocolor) completely lack viral capsids and anti-PRV immunogold particles, in contrary to PRV-infected neurons (cyan pseudocolor). Confocal pictures (second panel) showing that microglia do not express the immediate early GFP marker, in contrast to neurons, where a strong GFP expression can be seen in both the cell bodies and neurites. Scale bars: 1 μm on TEM images, 50 μm, 10 μm on the confocal panels.

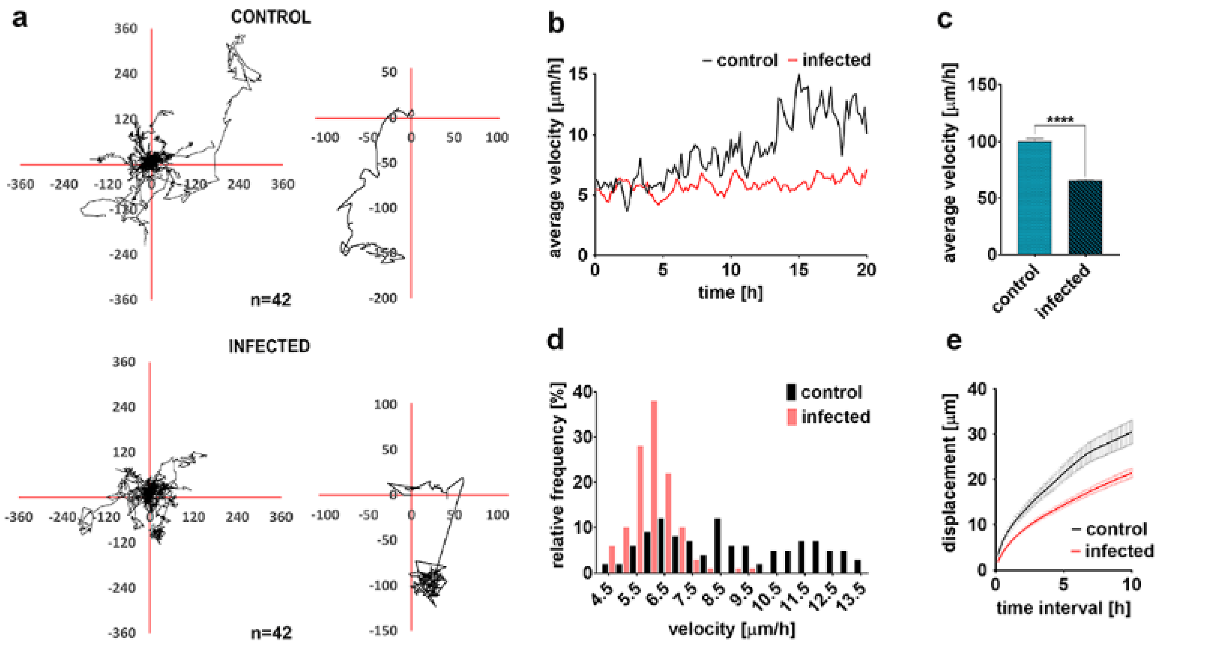

Supplementary Figure 3. Virus infection triggers the recruitment and phagocytic activity of microglia in primary neuronal cell cultures. a) Trajectories of microglial cells over a 24-hour imaging period in control and infected primary neuronal cultures. Individual cell trajectories were centered at the origin. Insets show typical trajectories of individual cells. b) Time-dependent average velocities of microglial cell populations in control (n=43) or infected (n=155) neuronal cultures. c) Average velocities of microglial cells over 24 hours are significantly lower in infected neuronal cultures ($p<0.0005$, error bars correspond to s.e.m). d) Frequency distribution of time-dependent average velocities of microglial populations in control or infected neuronal cultures. e) Average displacement of microglial cells in various time intervals of migration in control or infected neuronal cultures. Error stripes correspond to s.e.m.

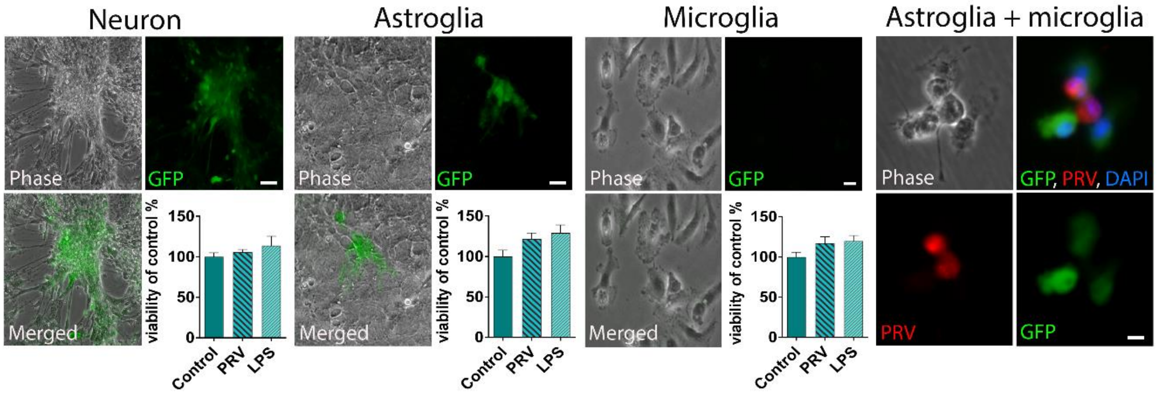

Supplementary Figure 4. Pseudorabies virus does not induce productive infection in microglial cells *in vitro*. In primary neuronal, astroglial or microglial cell cultures (bottom panels) infection with Bartha-DupGreen (BDG) was induced 24 hours prior to the assessment of infection-induced GFP expression or the cell viability (MTT) assay. Note that productive infection does not develop in microglia while it can be seen both in neurons and astrocytes. Viability of neurons, astrocytes and microglia is not reduced upon PRV infection or treatment with bacterial lipopolysaccharide (LPS). Data are expressed as mean ± s.e.m.. Scale bars: 50 μm, 15 μm.

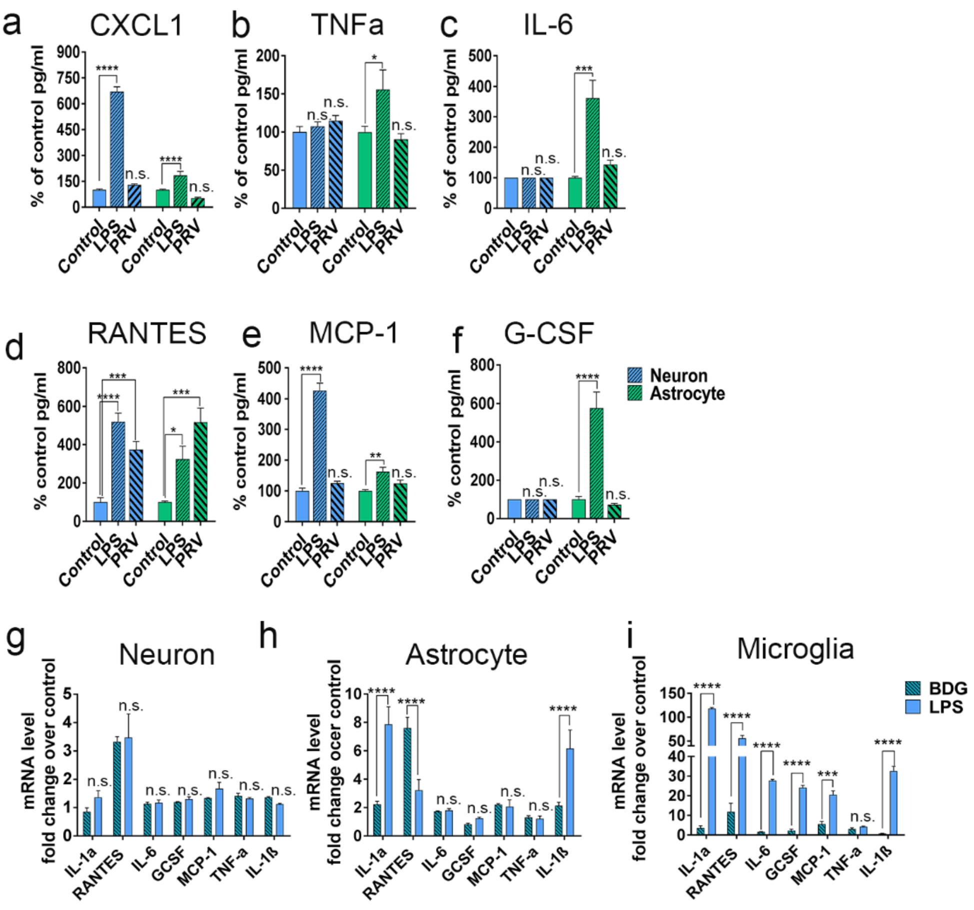

Supplementary Figure 5. Changes in peptide and mRNA levels of inflammatory cytokines and chemokines in response to virus infection in astroglial, neuronal and microglia cell cultures. Cytokine levels were measured in the conditioned medium of astroglial and neuronal cell cultures by cytometric bread array after LPS treatment or PRV infection. Samples were collected 24 hours after infection. Cell homogenates were collected for qPCR measurement. a-f, one-way ANOWA, followed by Tukey's post hoc test, a,****P<0.0001 Cont vs LPS, b, *P<0.05 Cont vs LPS, c, ***P<0.0001 Cont vs LPS, d, ****P<0.0001 Cont vs LPS, ***P<0.0001 Cont vs LPS, Cont vs PRV, *P<0.05 Cont vs LPS, e, ****P<0.0001 Cont vs LPS, **P<0.01 Cont vs LPS, f, ****P<0.0001 Cont vs LPS, g-i, 2way ANOVA followed by Sidak's multiple comparisons test. h, ****P<0.0001 BDG vs LPS, i, ****P<0.0001 BDG vs LPS, ***P<0.0001 BDG vs LPS.

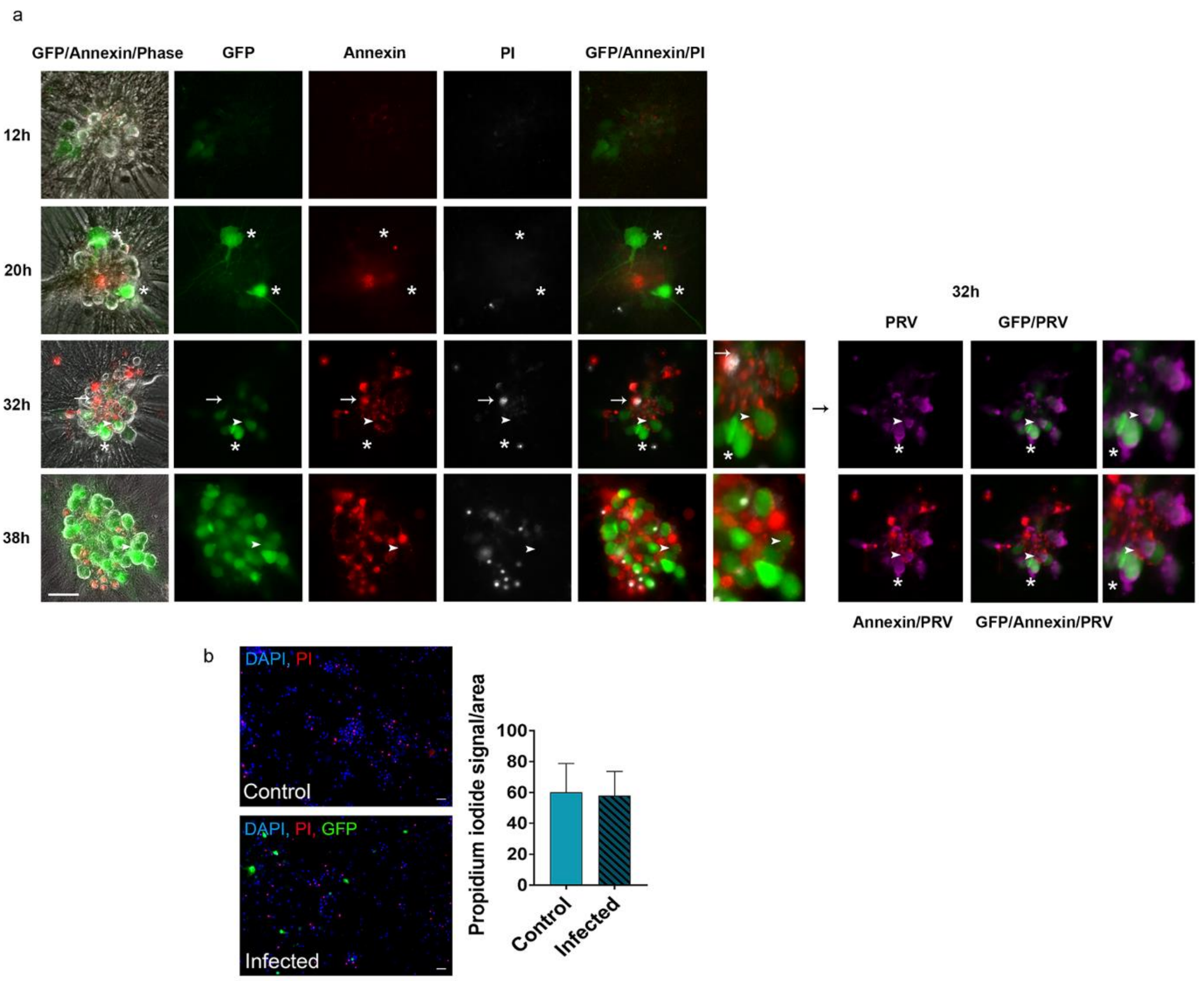

Supplementary Figure 6. Infected neurons remain morphologically intact until late stages of virus infection *in vitro*. a) Time course of BDG infection in primary neuronal cell cultures. Neuronal cultures were infected with a low titre of BDG to induce slow propagation of infection enabling the investigation of apoptotic and dead cells by using neuron-specific live annexin V staining and Propidium iodide (PI), respectively. Infected neurons show neither annexin V binding nor PI uptake until several hours following the appearance of the immediate early reporter protein, GFP. Neuron-specific live annexin V staining was first detected in neurons with decaying GFP expression. Note GFP^{high}/$annexin^{-}$ (asteriks) and GFP^{low}/$annexin^{+}$ (arrowheads) cells. PI entered cells only with intense annexin V labelling and little or no detectable GFP expression (arrow) indicating advanced infection. Immunostaining of the same cell culture after 32h post-infection revealed viral structural proteins (PRV) both in the GFP^{high} and GFP^{low} cells, indicating that both immediate-early reporter protein expression and viral protein expression precede the onset of infection-induced neuronal apoptosis. The images were captured with a Zeiss Axiovert epifluorescent microscope. Scale bar: 50 µm. b) Propidium iodide (PI, red) uptake was not altered in infected neurons 16 hours after infection. This particular PI uptake experiment was performed in parallel cultures to those used for HPLC analysis (Fig4.a,b). Scale bar: 50 µm.

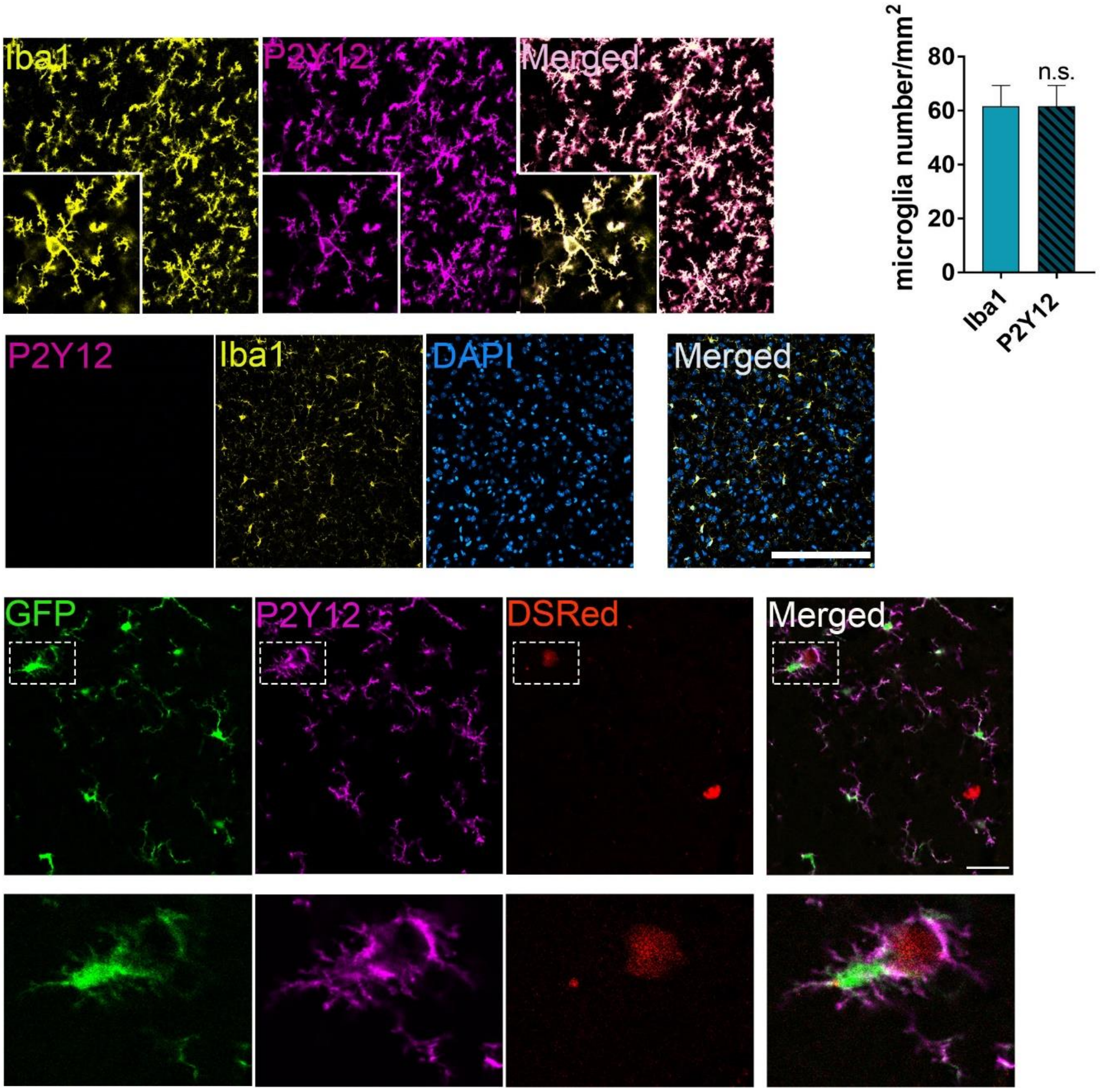

Supplementary Figure 7. P2Y12 is expressed by Iba1-positive microglia in the brain. P2Y12 immunofluorescence precisely identifies microglia in the brain that are co-expressing the microglia/macrophage marker Iba1. Note that virtually all microglia are P2Y12-positive. P2Y12 immunostaining is absent in P2Y12$^{-/-}$ mice. P2Y12 immunofluorescence co-localises with Iba1 and GFP in Cx3Cr1$^{+/gfp}$ microglia recruited to infected neurons expressing the immediate early marker, DSRed. Data are expressed as mean ± s.e.m. n.s.- not significant, Scale bar: 100 μm (top), 50 μm (bottom).

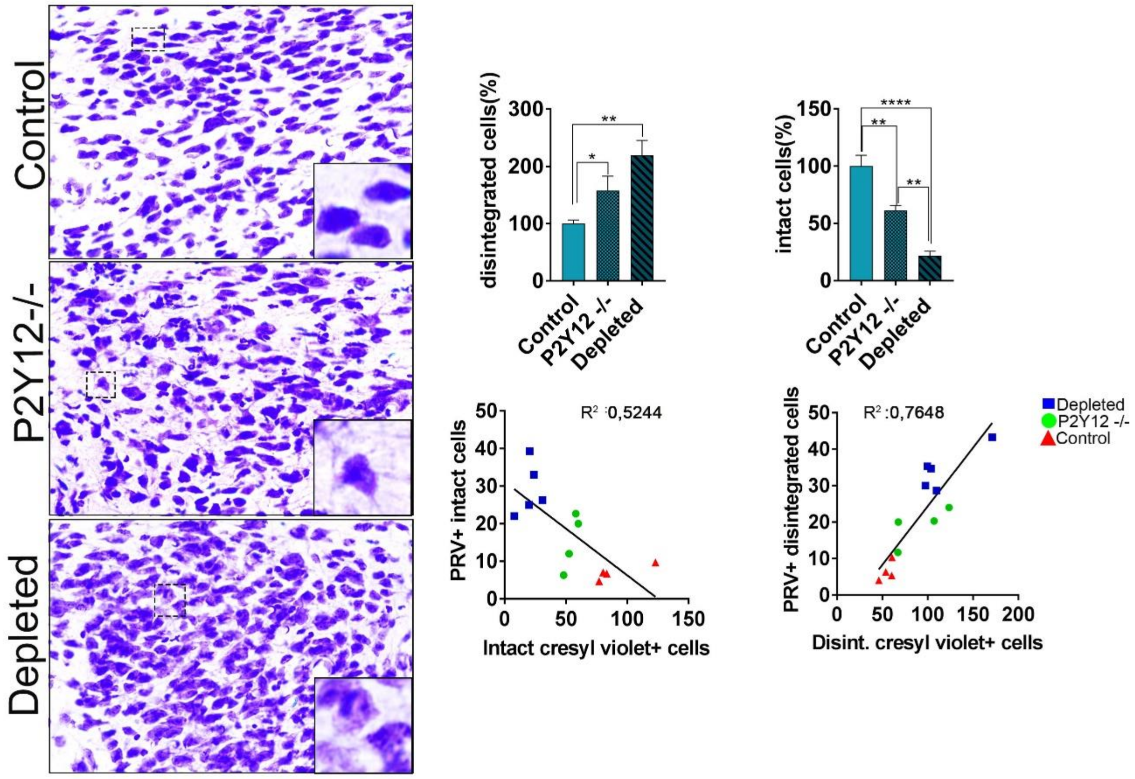

Supplementary Figure 8. The amount of disintegrated and dead cells is markedly increased in the brain in microglia-depleted and P2Y12$^{-/-}$ mice. a) Cresyl violet staining was performed on brain sections derived from control, P2Y12$^{-/-}$ and microglia-depleted mice 6 days after BDG infection. The number of disintegrated cells were increased (b), while the number of intact cells were markedly decreased (c) in the hypothalamus (PVN) of P2Y12 $^{-/-}$ and microglia-depleted mice compared to control animals. d) The number of morphologically intact cells (based on cresyl violet staining) show negative correlation with the number of intact, PRV-immunopositive cells (as presented in Fig.2a). e) The number of disintegrated, PRV-positive cells in Fig.2a correlates with morphologically damaged cells based on cresyl violet staining. b,c, one-way ANOWA, followed by Tukey's post hoc test, b, *P<0.05 Control vs P2Y12 $^{-/-}$, **P<0.01 Control vs Depleted. c, one-way ANOWA, followed by tukey's post hoc test, **P<0.01 Control vs P2Y12 $^{-/-}$; P2Y12 $^{-/-}$ vs Depleted, ****P<0.0001 Control vs Depleted. Scale bar: 10 µm.

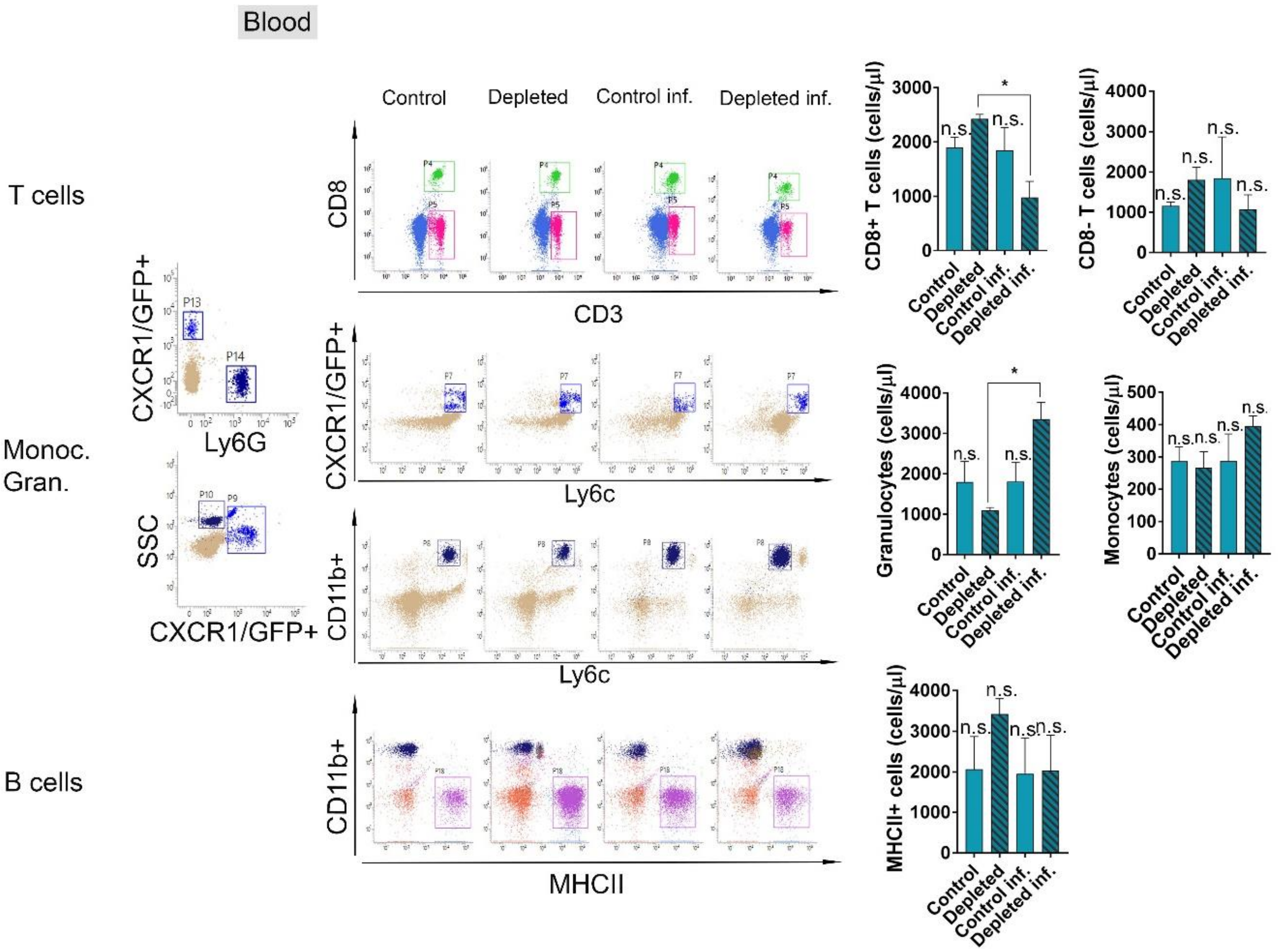

Supplementary Figure 9. Selective depletion of microglia does interfere with main blood cell populations. Cx3Cr1$^{+/gfp}$ mice were fed a PLX5622 chow diet for 21 days to deplete microglia. On the 16th day of the diet, a group of mice were injected with Bartha-DupGreen (BDG). Blood samples were collected from control mice and from mice 5 days after virus infection with or without microglia depletion, and labelled with mixtures of specific antibodies against leukocyte markers followed by flow cytometric analysis. Total blood cell counts were calculated by using 15 µm polystyrene microbeads (Polybead Microspheres, 18328-5). The number of CD8$^-$ (P5 gate) T lymphocytes, CD11b$^+$ Ly6c$^+$ SSC^{high} Ly6G$^+$ granulocytes (P8 gate), CD11b$^+$ Ly6c^{high} Cx3Cr1$^+$ (Ly6G$^-$) monocytes (P7 gate) and MHCII$^+$ CD11b$^-$ B cells (P18 gate) were no different between control, depleted, control infected and depleted infected animals. In association with the profoundly increased CNS infection in microglia depleted mice, numbers of CD3$^+$ CD8$^+$ T lymphocytes (P4 gate) and CD11b$^+$ Ly6c$^+$ SSC^{high} Ly6G$^+$ granulocytes (P8 gate) showed significant difference between depleted and depleted virus infected animals. Data are expressed as mean ± s.e.m. one-way ANOVA, n=4. n.s. - not significant.

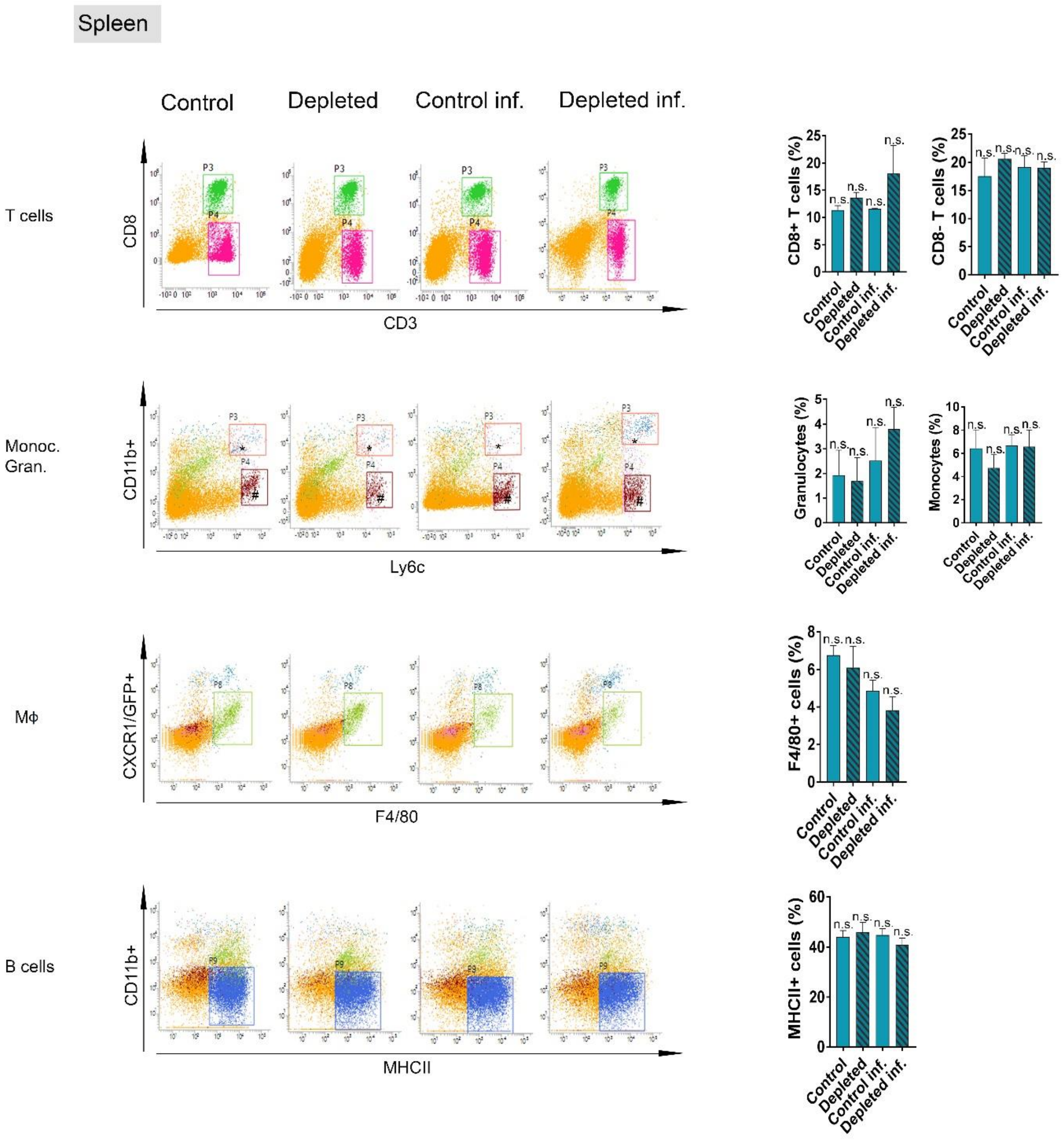

Supplementary Figure 10. Selective depletion of microglia does interfere with leukocyte numbers in the spleen. Cx3Cr1$^{+/gfp}$ mice were fed a PLX5622 chow diet for 21 days to deplete microglia. On the 16th day of the diet mice were injected with Bartha-DupGreen (BDG). Blood samples were collected from control mice and from mice 5 days after PRV infection, and labelled with mixtures of specific antibodies against leukocyte markers followed by flow cytometric analysis. Numbers of CD8^{+} T cells (P3 gate), CD8^{-} T cells (P4 gate) and MHCII^{+} B cells (P9 gate) were not different between control, depleted, control infected and depleted infected animals. Spleen macrophages (Cx3Cr1-gfp^{+} F4/80^{+} cells, P8 gate), granulocytes (P3* gate, CD11b^{+}, Ly6c^{+}, SSC^{high} cells) and monocytes (P4# gate, CD11b$_{low}$ Ly6c^{vhigh} cells) were not different between control, depleted, control infected and depleted infected mice. Data are expressed as mean ± s.e.m. One-way ANOVA, n=4.

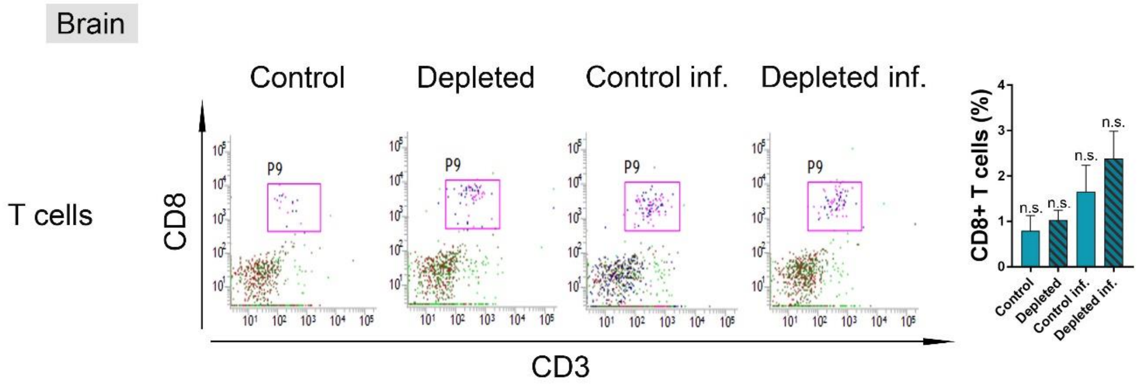

Supplementary Figure 11. Virus infection and selective manipulation of microglia do not alter the numbers of T lymphocytes in the brain. Cx3Cr1$^{+/gfp}$ mice were fed a PLX5622 chow diet for 21 days to deplete microglia. On the 16th day of the diet mice were injected with Bartha-DupGreen (BDG), to assess the role of microglia in inflammatory cell recruitment into the brain. Brain cells were isolated and T lymphocytes labelled with CD8, CD3 antibodies followed by flow cytometric analysis. Numbers of CD8+ T cells (P9 gate) were not different between control, depleted, control infected and microglia depleted infected animals. Data are expressed as mean ± s.e.m. One-way ANOVA, n=4.

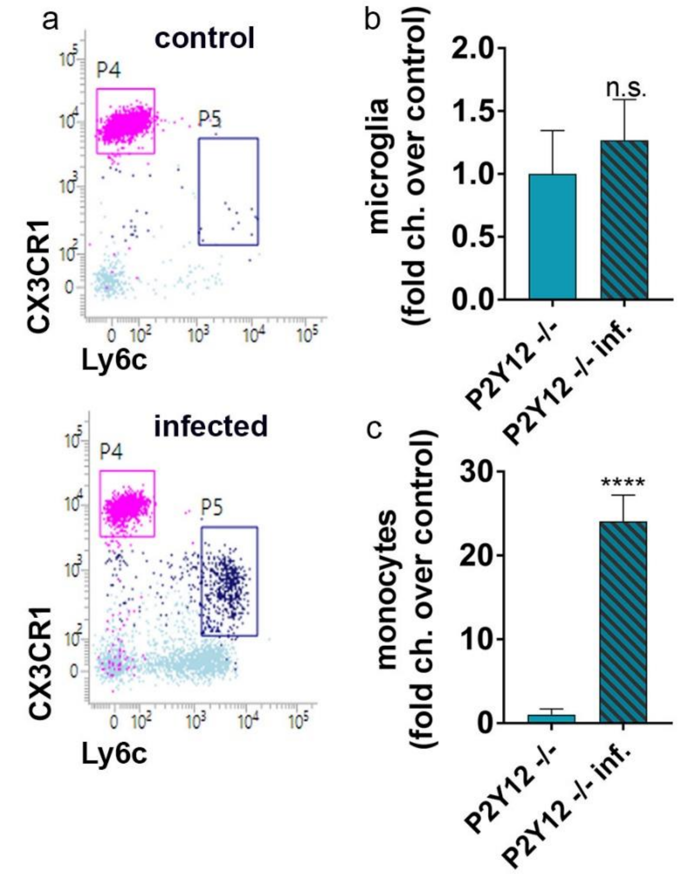

Supplementary Figure 12. Deficiency for P2Y12 does not influence infiltration of leukocytes into the brain upon virus infection. a, Flow cytometric dot plots showing infiltrating monocytes (P5) in the brain. **b,** Microglial numbers do not change in response to viral infection compared to control conditions. **c,** In response to viral infection monocytes infiltrate into the brain in P2Y12 -/- mice. Data expressed as mean±s.e.m. **b,c,** n.s. not significant **c,** ****p<0.0001 n=10 unpaired t-test.

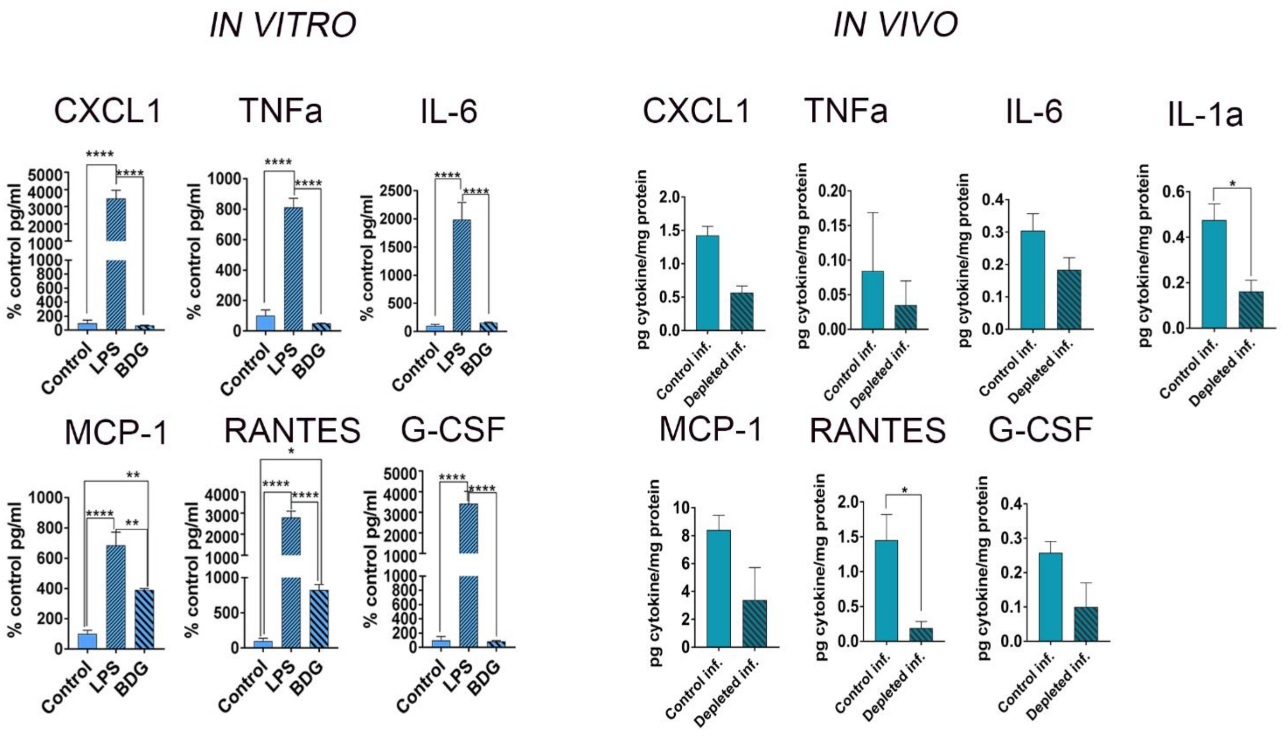

Supplementary figure 13. Changes in inflammatory cytokines and chemokines in response to virus infection and selective elimination of microglia. Left, *in vitro* data: Cytokine and chemokine levels were measured in the conditioned medium of microglial cell cultures by cytometric bead array after LPS treatment or exposure to PRV. Samples were collected 24 hours after infection. Right, *in vivo* data: Cytokine and chemokine levels were measured from homogenates of hypothalamic brain tissues of control and microglia-depleted mice infected with PRV in a retrograde transneuronal manner, 5 days prior to sample collection. All measurements were performed by cytometric bead array. One-way ANOVA, followed by Tukey's post-hoc test (left) or unpaired t test (right). n=5-6. Data are expressed as mean ± s.e.m.

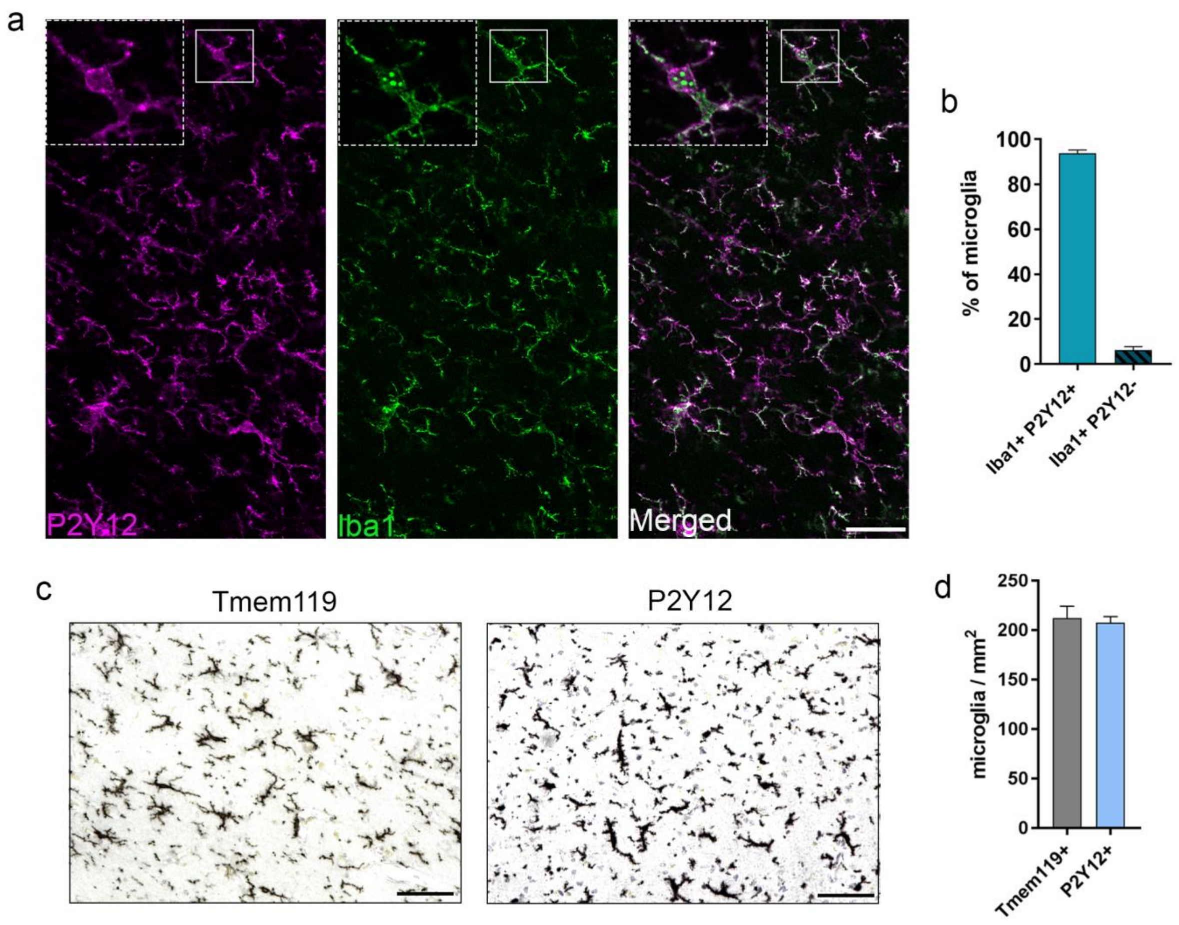

Supplementary figure 14. Microglia express P2Y12 in the human brain. a) Co-localization of microglial P2Y12 and Iba1 on perfusion-fixed, free-floating brain sections (frontal cortex). b) Proportion of P2Y12-positive microglia among Iba1+ cells. c) Immunohistochemistry showing Tmem119-positive and P2Y12-positive microglia in paraffin-embedded brain sections (frontal cortex). d) Quantification of Tmem119-positive and P2Y12-positive microglia in the brain. All samples are derived from patients with no known neurological disease.

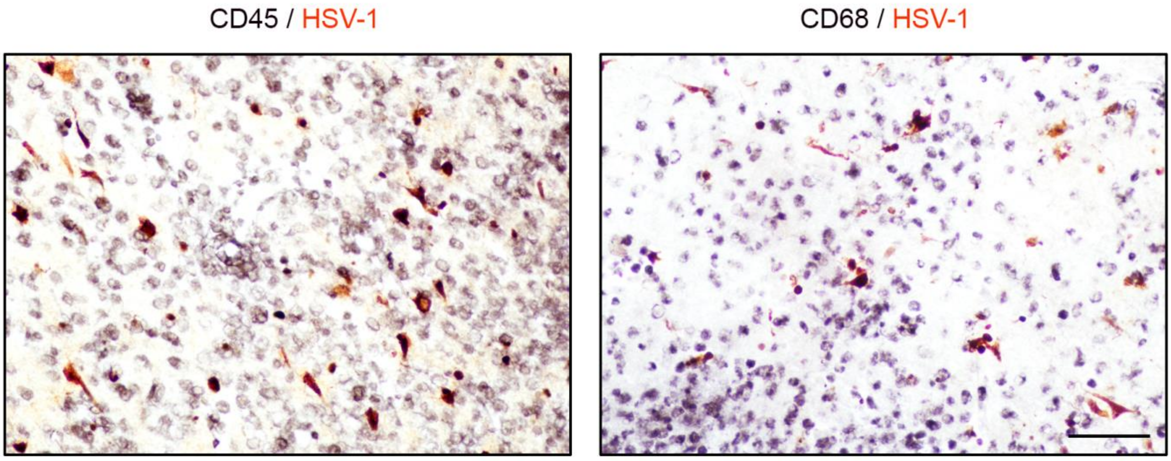

Supplementary figure 15. Advanced HSV-1 infection is associated with marked neuroinflammatory responses in the human brain. Recruitment of numerous CD45-positive cells and CD68-positive brain macrophages is seen at sites of advanced virus infection characterized by large number of HSV-1-positive cells. Scale bar: 50µm.

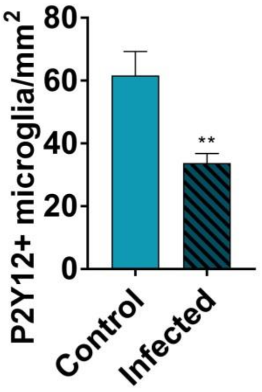

Supplementary Figure 16. Numbers of Cx3Cr1$^{+/gfp}$ microglia are reduced in areas of advanced PRV infection (heavy virus load) compared to that seen in uninfected (control) mice. Unpaired t test, n=5-6. Data are expressed as mean ± s.e.m.

	Nr.	Age (y)	Sex	HSVE survival	Assessed region	Co-pathology	Cause of death	Post-mortem delay	Fixation time
**cases**	1	35	female	21 years	temporal lobe	none	bronchopneumonia	<24 h	8 weeks
	2	66	female	16 days	temporal lobe	none	acute encephalitis / brain edema / herniation	<24 h	8 weeks
	3	65	female	9 days	temporal lobe	none	acute encephalitis / brain edema / herniation	<24 h	3 weeks
	4	41	female	24 months	temporal lobe	none	bronchopneumonia	<24 h	4 weeks
	5	24	male	10 days	temporal lobe	none	acute encephalitis / brain edema/herniation	62h	3 weeks
**controls**	Sko 13	60	female	N.A.	temporal lobe	chronic bronchitis	respiratory arrest	<24 h	2-3h perfusion and overnight post-fixation
	Sko 16	72	male	N.A.	temporal lobe	acute bronchitis	respiratory arrest	<24 h	2-3h perfusion and overnight post-fixation

Supplementary Table 1. Patient characteristics and tissue processing information for post-mortem human brain tissues

Supplementary Video legends

Supplementary Video 1. *In vivo* two-photon imaging shows PRV-Bartha-DupDSRed (BDR) positive neuron contacted by CX3CR1$^{+/gfp}$ microglia. Note that the recruitment of microglia, including the displacement of the cell bodies takes place within 3 hours. The video corresponds to Figure 2 c, panel.

Supplementary Video 2. 3D-reconstruction from deconvolved confocal stack showing a recruited microglia (green) engulfing a PRV-positive neuronal cell body (cyan) with CD68-positive phagolysosomes (magenta) within the microglia, arranged around the infected neuron. The thickness of the reconstructed volume is 50 μm, for details, see Figure 2.

Supplementary Video 3. Time-lapse recording of a neuron-microglia co-culture in the absence of infection. Note that the CX3CR1$^{+/gfp}$ microglia cell (green) neither perturbs neuronal process outgrowth nor disrupts an existing process. The time-lapse covers a 12h period. Phase-contrast and fluorescent images were recorded simultaneously every 10 min. The video corresponds to Figure 3 a, upper panel.

Supplementary Video 4. Time-lapse recording of a neuron-microglia co-culture in the absence of infection. Note that the CX3CR1$^{+/gfp}$ microglia cell (green) slipping under neurons does not harm the dense network above. The time-lapse covers a 7h period. Phase-contrast and fluorescent images were recorded simultaneously every 10 min. The video corresponds to Figure 3 a, mid panel.

Supplementary Video 5. Time-lapse recording of a neuron-microglia co-culture after neurotrophic virus infection. Neurons were infected with BDL virus 2 hours prior the start of imaging. Note that GFP-expressing, microglial cells (green) flatten (become activated) and phagocytose compromised cells. The time-lapse covers a 40h period. Phase-contrast and fluorescent images were recorded simultaneously every 10 min. The video corresponds to Figure 3 a, bottom panel.

Supplementary Video 6. Time-lapse recording of an astroglia-microglia co-culture in the absence of infection. Note, that some of the CX3CR1$^{+/gfp}$ microglial cells (green) interact with multiple astrocytes. The time-lapse covers a 24h period. Phase-contrast and fluorescent images were recorded simultaneously every 10 min. Trajectory of a typical microglial cell is shown with spectral color codes changing from red to blue as time elapses. The video corresponds to Figure 3 b, upper panel.

Supplementary Video 7. Time-lapse recording of an astroglia-microglia co-culture after neurotrophic virus infection. Cells were infected with BDL virus 2 hours prior the the start of imaging. CX3CR1$^{+/gfp}$ microglial cells (green) intensely scan the compromised cells and phagocytose them. Trajectories of microglial cells are shown with spectral color codes changing from red to blue as time elapses. The time-lapse covers a 28h period. Phase-contrast and fluorescent images were recorded simultaneously every 10 min. The video corresponds to Figure 3 b, bottom panel.

Supplementary Video 8. Trajectories of P2RX7$^{-/-}$ microglial cells migrating for a 20 h period in untreated (left) or BDG virus infected (right) astroglial cultures. For better visibility 20 randomly selected individual trajectories were centered to start from the origin on each side and superimposed with color codes changing from red to blue as time elapses. Note the more localized migration pattern of microglial cells in virus-infected culture on the right.

Supplementary Video 9. Trajectories of P2RY12$^{-/-}$ microglial cells migrating for a 20 h period in untreated (left) or BDG virus infected (right) astroglial cultures. For better visibility 20 randomly selected individual trajectories were centered to start from the origin on each side and superimposed with color codes changing from red to blue as time elapses.

Supplementary Video 10. Phagocytic activity of wild type microglia. The flattened microglial cell phagocytoses two BDG virus infected (GFP-expressing) cells (green) within 24 hours. Phase-contrast and fluorescent images were recorded simultaneously every 10 min. The video corresponds to Figure 4 q.

Supplementary Video 11. Phagocytic activity of microglia with deficient purinergic signaling. Note that P2RX7 $^{-/-}$ microglia (left panel) readily phagocytose a BDG virus infected (GFP-expressing) cell (green) while the phagocytic activity of P2RY12$^{-/-}$ microglia (right panel) is abolished. The time-lapse covers a 12h period. Phase-contrast and fluorescent images were recorded simultaneously every 10 min. The video corresponds to Figure 4 q.

Supplementary Methods

Mice

Mice were bred and genotyped at the SPF unit of the Animal Care Unit of the Institute of Experimental Medicine (IEM HAS, Budapest, Hungary) as described earlier[12, 17]. Mice had free access to food and water and were housed under light-, humidity- and temperature-controlled conditions.

Selective elimination of microglia from the brain

PLX5622 was provided by Plexxikon Inc. (Berkeley, USA) and formulated by Research Diets (New Brunswick, USA) into an AIN-76A standard chow in 1200 p.p.m. (1200mg PLX 5622 in 1kg chow) concentration. Mice were fed PLX5622 for 3 weeks, to eliminate microglia from the brain. We could not observe any sign of physiological illness (alterations in food intake, weight, physical appearance) or behavioural changes (social interactions, exploration) during the diet period, in accordance with other studies[10, 17].

Neurotropic herpesvirus infection

Mice were randomly assigned to experimental groups and were injected either intraperitoneally or directly into the epididymal white adipose tissue with a genetically modified PRV-Bartha derivative, PRV-Bartha-Dup-Green (BDG)[3] to induce retrograde transsynaptic infection in the brain. To enable a defined, exclusively retrograde transsynaptic spread of infection *in vivo,* the neuroinvasiveness of BDG was modified by insertion of a GFP gene expression cassette to the putative antisense promoter (ASP) located at the repeated invert region of the virus. This construct also allows time-mapping the spread of infection in individual cells[3, 4, 7]. The activity of the strong immediate-early promoter/enhancer of the human cytomegalovirus is independent from the viral protein, enabling very early stages of infection to be detected by GFP expression. PRV genes encoding structural proteins are driven by late promoters, resulting in the appearance of the virus proteins in the late stage of the infection. In a set of studies, mice were infected with BDG on 16th day of PLX5622 diet to assess the effect of microglia depletion on central propagation of virus infection. For *in vivo* two-photon imaging, Cx3Cr1$^{GFP/+}$ mice were infected with PRV-Bartha-DupDsRed (BDR)[2] enabling the co-detection of infected neurons with microglia. The virus was grown in porcine kidney (PK)-15 cells to a titer of 6x10^{8} plaque-forming units (PFU)/ml. After virus injection (10 µl intraperitoneally or into the epididymal white adipose tissue), mice were let to survive for 6 or 7 days and were regularly monitored for neurobehavioral symptoms.

Tissue processing and immunostaining

Mice were transcardially perfused with saline followed by an ice-cold 4% PFA solution. Brains were post-fixed, cryoprotected and 25µm thick sections were cut. Fluorescent immunohistochemistry was performed on free-floating brain sections using a combination of goat anti-mouse Iba1 (1:500, # NB100-1028, Novus Biologicals), chicken anti-GFP tag (1:1000, #A10262, Invitrogen), rabbit anti-PRV (1:500, generous gift of Lynn Enquist), rabbit anti-mouse P2Y12 (1:500, # 55043A, AnaSpec), rat anti-mouse CD45 (1:250, #MCA1388, AbD Serotec), and rat anti-mouse CD68 antibodies (1:250 dilution, #MCA1957GA, AbD Serotec). Brain sections were blocked with 2% normal donkey serum and incubated with a mixture of primary antibodies overnight at 4C° which was followed by the cocktail of

appropriate secondary antibodies: donkey-anti-goat Alexa 594 (1:500, #A11058, Invitrogen), donkey anti-chicken Alexa 488 (1:500, #703-546-155, Jackson ImmunoResearch), or donkey-anti-rabbit Alexa 647 antibodies (1:400, #711-605-152, Jackson ImmunoResearch). Biotinylated tomato lectin (1:100, #L0651-1 mg, Sigma-Aldrich) was visualized by streptavidin Marina Blue or Alexa 594 conjugates (1:250, #S-11221, Invitrogen). Images were captured with a Nikon Ni-E C2+ confocal microscope, and image processing was done using the NIS Elements Viewer 4.20 software. Quantitative analysis was performed on 3 randomly selected fields within the region of interest for each brain section, on 3-3 serial coronal sections for given brain areas.

Super-resolution (STORM) microscopy

Free-floating brain sections were blocked with 2% normal donkey serum followed by immunostaining with rabbit anti-mouse P2Y12 antibody (1:500, #AS-55043A, AnaSpec) and donkey anti-rabbit Alexa 647 secondary antibody (1:400, #711-605-152, Jackson ImmunoResearch), chicken anti-GFP tag (1:1000, #A10262, Invitrogen) and donkey anti-chicken Alexa 488 (1:500, #703-546-155, Jackson ImmunoResearch), rabbit anti-PRV (1:500, gift of Lynn Enquist,) and donkey anti-rabbit Alexa 647 (1:400, #711-605-152, Jackson ImmunoResearch). Sections were mounted onto #1.5 thick borosilicate coverslips and covered with imaging medium[50] immediately before imaging. The imaging medium contained: 5% glucose, 0.1 M mercaptoethylamine, 1 mg ml−1 glucose oxidase, and catalase (1500 U ml−1, Sigma-Aldrich) in Dulbecco's PBS (Sigma-Aldrich). STORM imaging was performed for P2Y12 (stimulated by a 647 nm laser) by using a Nikon N-STORM C2+ super-resolution system that combines 'Stochastic Optical Reconstruction Microscopy' technology and Nikon's Eclipse Ti research inverted microscope to reach a lateral resolution of 20 nm and axial resolution of 50 nm[1, 9].

Immuno-electron microscopy

For combined immunogold-immunoperoxidase stainings, animals were perfused with 4% paraformaldehyde (PFA) and 0.1% glutaraldehyde in 0.1 M phosphate buffer (PB) pH 7.4 for 40 minutes and 50 μm thick sections were cut. Sections were washed in PB, treated with 0.5% sodium-borohydride for 5 minutes, and further washed in PB and TBS. This was followed by incubation in 1% human serum albumin (HSA, #A1653-5G, Sigma-Aldrich), diluted in TBS. Then the sections were incubated for 48 hours in the following solutions of primary antibodies diluted in TBS: either guinea-pig anti-Iba1 (1:500, #234 004, Synaptic Systems) or chicken anti-eGFP (in case of the $Cx3CR1^{GFP/+}$ mice, 1:1000, #A10262, ThermoFisher Scientific) was mixed with rabbit anti-PRV (1:500, from Lynn Enquist). To eliminate non-specific binding, the anti-PRV antibody solution was incubated with control brain sections for one day before use. After repeated washes in TBS, sections were treated with blocking solution (Gel-BS) containing 0.5% cold water fish skin gelatin (#900.033, Aurion, The Netherlands) and 0.5% HSA in TBS for 1 h. This was followed by 24 h incubation in the following solutions of secondary antibodies diluted in Gel-BS: 1.4 nm gold conjugated goat anti-rabbit Fab-fragment (1:200, #2004, NanoProbes) combined either with biotinylated donkey anti-guinea pig (1:500, #706-065-148, Jackson ImmunoResearch) or biotinylated goat anti-chicken (1:500, #BA-9010, Vector Laboratories). After intensive washes in TBS and 0.1 M PB sections were treated with 2% glutaraldehyde in 0.1 M PB for 15 minutes. This was followed by further washes in 0.1 M PB, TBS and incubation in ABC (1:300, Vectastain Elite ABC HRP Kit, #PK-6100, Vector Laboratories) diluted in TBS. Sections were washed in TBS, and tris-buffer (TB) pH 7.6, and the immunoperoxidase

reaction was developed using 3,3-diaminobenzidine as chromogen (DAB, #32750-25G-F, Sigma-Aldrich). After repeated washes in TBS and enhancement conditioning solution (ECS, #500.055, Aurion), gold particles were intensified using the silver enhancement solution (SE-EM, #500.044, Aurion) for 60 minutes at room temperature. After subsequent washes, sections were treated with 0.5 % osmiumtetroxide in PB for 20 minutes. Then sections were dehydrated in ascending ethanol series and acetonitrile, and embedded in epoxy resin (Durcupan, #44610-1EA, Sigma-Aldrich). During dehydration sections were treated with 1% uranylacetate in 70% ethanol for 20 minutes. After polymerization, 70 nm thick sections were cut on a Leica EM UC6 ultramicrotome (Nussloch, Germany), and picked up on formvar-coated single-slot copper grids. The sections were examined using a Hitachi H-7100 electron microscope (Tokyo, Japan) and a side-mounted Veleta CCD camera (Olympus Soft Imaging Solutions).

Correlated confocal laser-scanning microscopy, electron microscopy and electron tomography

Before the immunofluorescent staining, the 50 µm thick brain sections from BDG-injected Cx3CR1$^{GFP/+}$ mice were washed in PB, treated with 0.5% sodiumborohydride for 5 minutes, and further washed in PB and TBS. This was followed by blocking for 1 hour in 1% human serum albumin (HSA). After this, sections were incubated in mixtures of primary antibodies: chicken anti-eGFP (1:4000, #A10262, ThermoFisher Scientific), rat-anti-mouse CD68 (1:250, #MCA1957GA, AbD Serotec) and rabbit anti-PRV (1:500, gift of Lynn Enquist). To eliminate non-specific binding, the anti-PRV antibody solution was incubated with control brain sections for one day before use. After repeated washes in TBS, sections were treated with blocking solution containing 0.5% cold water fish skin gelatin and 0.5% HSA in TBS for 1 h. After incubation, sections were washed in TBS, and incubated overnight at 4 °C in the mixture of biotinylated goat-anti-chicken (1:500, #103-065-155, Jackson ImmunoResearch), Alexa 594 conjugated donkey-anti-rat (1:500, #A21209, Invitrogen) and Alexa 647 conjugated donkey-anti-rabbit (1:500, #711-605-152, Jackson ImmunoResearch) diluted in GelBS. Secondary antibody incubation was followed by washes in TBS and PB. Sections were mounted in PB, coverslipped, sealed with nail-polish. Immunofluorescence was analysed using a Nikon Eclipse Ti-E inverted microscope (Nikon Instruments Europe B.V., Amsterdam, The Netherlands), with a CFI Plan Apochromat VC 60X water immersion objective (NA: 1.2) and an A1R laser confocal system. We used 488, 561 and 642 nm lasers, and scanning was done in line serial mode. Image stacks were obtained with NIS-Elements AR software with a pixel size of 50x50 nm in X-Y, and 150 nm Z-steps. Stacks were deconvolved using Huygens Professional software (www.svi.nl), and 3D-reconstruction was performed using the IMOD software package[16]. After imaging, sections were washed 0.1 M PB, TBS and incubated in ABC (1:300, Vectastain Elite ABC HRP Kit, #PK-6100. Vector Laboratories) diluted in TBS. Sections were washed in TBS, and tris-buffer (TB) pH 7.6, and the immunoperoxidase reaction was developed using 3,3-diaminobenzidine as chromogen. After subsequent washes, sections were treated with 0.5 % osmiumtetroxide in PB for 20 minutes. Then sections were dehydrated in ascending ethanol series and acetonitrile, and embedded in epoxy resin. During dehydration sections were treated with 1% uranylacetate in 70% ethanol for 20 minutes. After polymerization, 70 or 150 nm thick sections were cut on an ultramicrotome, and picked up on formvar-coated single-slot copper grids. The sections were examined using a Hitachi H-7100 electron microscope and a side-mounted Veleta CCD camera. For the electron tomographic investigation, we used the 150 nm thick sections. Grids were put on drops of 10% HSA in TBS for 10 minutes, dipped in distilled water (DW), put on drops of 10 nm gold conjugated Protein-A (#AC-10-05-05, Cytodiagnostics) in DW (1:3), and washed in DW. Finally, we deposited 5-5 nm thick layers of carbon on both sides of the grids. Electron tomography was performed using a Tecnai T12 BioTwin electron microscope equipped with a computer-controlled precision stage and an Eagle™ 2k CCD 4

megapixel TEM CCD camera. Acquisition was controlled via the Xplore3D software (FEI). Regions of interest were pre-illuminated for 4-6 minutes to prevent further shrinkage. Tilt series were collected at 2 degree increment steps between -65 and +65 degrees at 120 kV acceleration voltage and 23000x magnification with -1.6 – -2 µm objective lens defocus. Reconstruction was performed using the IMOD software package. Isotropic voxel size was 0.49 nm in the reconstructed volumes. Segmentation has been performed on the virtual sections using the 3Dmod software.

Immunohistochemistry and immunofluorescence on post-mortem human brain samples

To investigate microglia recruitment in response to neurotropic virus infection in the human brain, formalin-fixed paraffin-embedded (FFPE) post-mortem brain tissues from five patients aged 42-66 years were analyzed (ethical approval ETT-TUKEB 62031/2015/EKU and TUKEB 34/2016) with Herpes simplex type 1 encephalitis (HSVE1) confirmed by PCR and immunohistochemistry. Tissue samples from two additional patients with no known neurological disease have been used as controls. Control samples were perfuse-fixed with Zamboni fixative (4% PFA, 15 % PIC) and postfixed overnight in the same solution. 4-6µm thick brain sections were cut and samples were mounted on gelatin-coated slides. After deparaffinisation, HIER (Novocastra Epitope Retrieval Solution pH9, Leica Biosystems) and peroxidase blocking (in 1% H2O2 solution) was carried out. Sections were blocked with 2.5% Normal Horse serum (#S-2012, Vector Laboratories) and were incubated with rabbit anti-HSV I (#361A-14, Cell Marque), rabbit anti-P2Y12 (#AS-55043A, AnaSpec, Inc.), rabbit-anti-Tmem119, mouse anti-CD68, mouse anti-CD45, mouse anti-CD3, mouse anti-CD20 and mouse anti-CD15 antibodies. For signal amplification ImmPRESS anti-rabbit HRP Kit (#MP-7401, Vector Laboratories) and for visualisation either ImmPACT NovaRED HRP substrate (for detection of HSV I) or DAB-Ni HRP substrate (for detection of other markers, #SK-4805, Vector Laboratories) was used. Representative pictures were taken on 20x magnification using a Nikon Ni-E C2+ microscope. To assess the proportion of P2Y12-positive microglia, P2Y12 and Iba1 double immunofluorescence has been performed on 50 µm thick free-floating brain sections. Images were captured using a Nikon Eclipse Ti-E inverted microscope (Nikon Instruments Europe B.V., Amsterdam, The Netherlands), with a CFI Plan Apochromat VC 60X water immersion objective (NA: 1.2) and an A1R laser confocal system.

Two-photon imaging

To assess microglia recruitment to infected neurons in the mouse brain in real-time, Cx3CR1$^{GFP/+}$ mice were i.p. injected with 10µl of the BDR virus. 7 days after virus injection, cranial window surgery was performed on anaesthetized (using 3% isoflurane for induction, 1.5-2% during surgery) mice and a circular craniotomy (3 mm diameter) was made above motor cortex (Bregma – 0,82); on the right hemisphere. A custom made aluminium head plate was fixed to the skull using Cyanoacrylate glue. During skull opening the place of craniotomy was washed continuously with cold Ringer solution. The craniotomy was covered with a circular cover glass and dura mater remained intact under the glass to ensure that microglial activation is not induced[17]. The cover glass was fixed with Paladur mixed Cyanoacrylate.

Measurements were performed on a Femto2D-DualScanhead microscope (Femtonics Ltd., Hungary) coupled with a Chameleon Ultra II laser (Coherent, Santa Clara, USA) [5, 13]. The wavelength of the laser was set to 980nm to measure DsRed and GFP signal simultaneously. Excitation was delivered to the sample, and the fluorescent signal was

collected using a CFI75 LWD 16XW/0.8 lens (Nikon, 16x, NA 0.8) and then separated using a dichroic mirror (700dcxru) before the two channel detector unit, which was sitting on the objective arm (travelling detector system) as described in detail earlier[5, 13]. The dichroic mirror and emission filters (490-550 nm for the green and 570-640 nm for the red channel) was purchased from Chroma Technology Corp. (Vermont, USA). Data acquisition was performed by MES softver (Femtonics Ltd.) A Z-stack from of 32 images (800x800 pixel, field-of-view=210x210 µ, Z stack contained 12 image planes with 4.6 µm step size (range= 80-135 µm from pial surface) was made at every 3 minutes. Two-photon image sequences were exported from MES and analysed using ImageJ.

Primary neuronal cultures

Primary cultures of embryonic cortical cells were prepared from CD1 mice on embryonic day 15[6]. Cells were seeded onto poly-L-lysine–coated (#P1524, Sigma-Aldrich) 24-well tissue culture plates at 3×10^5 cells/well density and grown in NeuroBasal medium (#21103-049, ThermoFisher Scientific) supplemented with 5% fetal bovine serum (FBS, #F7524, Sigma), B27 (#17504-044, ThermoFisher Scientific), Glutamax (#35050061, ThermoFisher Scientific), gentamicin (40 µg/ml, Sandoz), amphotericin B (2.5 µg/ml, #A2411, Sigma-Aldrich). Cytosine-arabinofuranoside (CAR, 5 µM, #C6645, Sigma-Aldrich) was added to the cultures 48-72 h after plating to limit glia growth, and then one third of the culture medium was changed to NeuroBasal medium supplemented with B27 without FBS every 3–4 day thereafter. Cells were cultivated for 6-8 days at 37°C in 5% CO_2, 95% air atmosphere until further measurements.

Astroglia and microglia cell cultures

Astroglia/microglia mixed cell cultures were prepared from the whole brains of CD1, C57BL/6J, $CX3CR1^{GFP/+}$, $P2X7^{-/-}$ or $P2Y12^{-/-}$ newborn (P0-P1) mouse pups, as described earlier[15]. In brief, meninges were removed and the tissue pieces were subjected to enzymatic dissociation, using 0.05% w/v trypsin (#T4549, Sigma Aldrich) and 0.05% w/v DNase (#DN-25, Sigma-Aldrich) for 10 minutes at room temperature. The cells were plated onto poly-L-lysine (#P1524, Sigma-Aldrich) coated plastic surfaces and were grown in Minimal Essential Medium (#M2279, Sigma-Aldrich) supplemented with 10% FBS (#10500, Gibco), 4 mM glutamine (#G3126, Sigma-Aldrich), 40 µg/ml gentamycin (Sandoz) and amphotericin B (2.5 µg/ml, #A2411, Sigma-Aldrich) in humidified air atmosphere containing 5% CO2, at 37°C. The culture medium was changed on the first two days and every third day afterwards. Cultures reaching confluency at ~DIV7 were harvested by trypsinization and re-plated onto pLL coated glass coverslips or into petri dishes, according to the actual experimental design. Secondary astrocytic cultures reaching confluency and displaying mosaic-like pattern were infected with PRV strains, as it follows. Microglial cells were isolated from 21-28 day old mixed cultures either by shaking or by mild trypsinization.

Neurotropic herpesvirus infection of primary neuronal and glial cultures

The neuronal or astroglia cultures were infected with either PRV-Bartha-Dup-Green (BDG) virus or PRV-Bartha-DupLac (BDL) at a final titer of 2.5×10^5 PFU/ml, as described earlier[11]. The multiplicity of infection (MOI) was ~0,17 PFU/cell. The cells were incubated with the virus containing medium for 1h, at room temperature. In order to remove infective virus particles from the culture medium we washed the cultures at least three times after the 1h viral exposure. For quantification of ATP derivatives released by or retained in control vs. infected cells, embryonic (E16) primary cortical neuronal cultures were used. The cells were infected at DIV9. 16 hours post-infection GFP expression was initiated in most cells, while the cell membrane integrity was not compromised and the overall viability was not reduced based on

MTT cell viability tests. The cell culture media and the cell fractions were collected 15 min after media change, as it is described below. For detemination of cytokine production as well as quantitative RT-PCR the following cultures and conditions were used.

Establishment of neuron/microglia and astroglia/microglia co-cultures

In cultures used for time-lapse recordings microglial cells isolated from mixed glial cultures were seeded on top of astrocytic or neuronal cell cultures in 10 000 cell/cm^2 density, immediately after the infection and the subsequent washing steps. If not CX3CR1$^{GFP/+}$ microglia were used, the cells were subjected to staining with the orange or red vital CellTracker dyes CMTMR/CMTPX (#C2927, #C34552, ThermoFisher Scientific) prior co-culture establishment (30min, 37°C).

Time-lapse microscopy

Time-lapse recordings were performed on a computer-controlled Leica DM IRB inverted fluorescent microscope equipped with a Marzhauser SCAN-IM powered stage and a 10x N-PLAN objective with 0.25 numerical aperture and 5.8 mm working distance. The microscope was coupled to an Olympus DP70 color CCD camera and a Zeiss Colibri LED epifluorescent illumination system. Cell cultures were kept at 37°C in humidified 5% CO_2 atmosphere in tissue culture grade Petri dishes in a stage-top incubator mounted on the powered stage of the microscope. Stage positioning, focusing, illumination and image collection were controlled by a custom-made experiment manager software on a PC. Phase contrast and epifluorescent images were collected consecutively every 10 minutes from several microscopic fields for durations up to 48 hours. Images were edited using NIH ImageJ software.

Cell motility data analysis

Cell tracking: Images were analyzed individually with the help of custom-made cell-tracking programs (G-track and Wintrack) enabling manual marking of individual cells and recording their position parameters into data files. At the magnification applied, the precision of this tracking procedure is estimated to be 10 µm, comparable to the average cell diameter (10-50 µm). In the following, the position of the ith cell at time *t* is denoted by $x_i(t)$.

Trajectories: Cell positions, $x_i(t)$, from several microscopic fields were recorded over 24 hours and each cell's trajectory was plotted. Subsequently, the starting positions of trajectories were aligned to the origin (x=0, y=0) and consecutive relative cell positions were plotted and superposed yielding groups of centered trajectories enabling the comparison of cell migration directionality. Trajectories in the monolayer cell cultures studied indicate a persistent random walk behavior with different velocity and directional persistence.

Displacement: The motion of individual cells is often evaluated in terms of average cell displacement, *d,* over a time period *t* as:

$$d^2(t) = \left\langle \left(X_i(t+t_0) - X_i(t_0) \right)^2 \right\rangle_i$$

where $X_i(t)$ denoting the center of cell *i* at time *t*, $\langle \cdot \rangle_i$ is an average over all possible cells, and t_0 is an arbitrary reference frame of the image sequence analyzed. The empirical *d(t)* curves indicate a persistent random walk behavior in monolayer cell cultures studied.

Average velocity: Average cell velocity was calculated directly from cell displacements in consecutive steps of cell tracking. The velocity, $v_i(t)$, of a given cell *i* at time *t* was calculated as

$v_i(t)=|x_i(t+ Dt) - x_i(t)| / Dt$

where D*t* is the difference of two consecutive steps of cell tracking and thus the time resolution data acquisition. To characterize the motility of an ensemble of cells, time-dependent average velocity *v(t)* was calculated as

$$v(t)=\frac{1}{N(t)}\sum_{i=1}^{N(t)}v_i(t)$$

where the summation goes over each *N(t)* cell being in the cell population. Average velocity, *v*, was calculated by averaging *v(t)* over all time steps of cell tracking as

$$v=\frac{1}{K}\sum_{t=\Delta t}^{K\cdot\Delta t}v(t)$$

where K is the number of time steps of tracking.

Cytokine measurement from media of primary cell cultures

Concentrations of IL-1α, IL-1β, TNF-α, IL-6, MCP-1, RANTES (CCL5), G-CSF and KC (CXCL1) were measured from conditioned media of primary neuronal, astroglial and microglial cell cultures by using cytometric bead array (CBA) Flex Sets (all from BD Biosciences, #560157, #560232, #558299, #558301, #558342, #558345, #560152, #558340, respectively). Measurements were performed on a BD FACSVerse machine and data were analysed using an FCAP Array software (BD Biosciences) as described earlier[8]. The cytokine levels of conditional media were corrected for total protein concentrations of the samples measured by Bradford Protein Assay Kit (#50000201, Bio-Rad Laboratories).

Total RNA isolation and quantitative RT-PCR

For total RNA isolation, cell culture samples were homogenized in 500 µl TRI Reagent and isolation was performed using Tissue Total RNA Mini Kit according to the manufacturer's instructions. To eliminate genomic DNA contamination, DNase I treatment was introduced (1 U/reaction, reaction volume: 50 µl). Sample quality control and the quantitative analysis were carried out by NanoDrop. cDNA synthesis was performed with the High Capacity cDNA Reverse Transcription Kit according to the manufacturer's instructions. The chosen primer sequences used for the comparative C_T experiments were verified with the Primer Express 3.0 and Primer-BLAST software. The sequences were as follows:

**GAPDH**	fw TGA CGT GCC GCC TGG AGA AA, rev AGT GTA GCC CAA GAT GCC CTT CAG
**IL-1α**	fw CCA TAA CCC ATG ATC TGG AAG AG, rev GCT TCA TCA GTT TGT ATC TCA AAT CAC
**TNF-α**	fw CAG CCG ATG GGT TGT ACC TT, rev GGC AGC CTT GTG CCT TGA
**MCP-1**	fw CCAGCACCAGCACCAGCCAA, rev TGGATGCTCCAGCCGGCAAC
**RANTES**	fw CAGCAGCAAGTGCTCCAATCTT, rev TTCTTGAACCCACTTCTTCTCTGG
**IL-6**	fw CTCTGCAAGAGACTTCCATCC, rev AGTCTCCTCTCCGGACTTGT
**G-CSF**	fw TGCCCAGAGGCGCATGAAGC, rev GGGGAACGGCCTCTCGTCCT

The primers (purchased from Invitrogen) were used in real-time PCR reaction with Fast EvaGreen qPCR Master Mix (Biotium, USA) on a StepOnePlus (Applied Biosystems) instrument. The gene expression was analyzed using the StepOne 2.3 program (Applied Biosystems). Amplicons were tested by Melt Curve Analysis on StepOnePlus instrument. Experiments were normalized to *gapdh* expression.

Quantification of nucleotides and adenosine

The adenine nucleotides (ATP, ADP, AMP) and adenosine (Ado) were determined in extracts from cells and culture media by using HPLC method. The medium (400 µl) was separated into a cold Eppendorf tube which contained 50 µl of homogenization solution. The cell layer was frozen with liquid nitrogen and extracted in 100 µl volume of ice-cold homogenization solution. The homogenization solution was 0.1 M perchloric acid that contained theophylline (as an internal standard) at 10 µM concentration. The cell extract was centrifuged at 3510 g for 10 min at 0-4°C and the pellet was saved for protein measurement. Perchloric anion from the supernatant was precipitated by 1 M potassium hydroxide, the precipitate was then removed by centrifugation. The culture media after the acid extraction was centrifuged as described above. The extracted purines were kept at -20°C until analysis. The adenine nucleotides and adenosine in extracts from cells and culture media were determined by online column switching separation using Discovery HS C18 50 x 2-mm and 150 x2-mm columns. The flow rate of the mobile phases ["A" 10 mM potassium phosphate, 0.25 mM EDTA "B" with 0.45 mM octane sulphonyl acid sodium salt, 8 % acetonitrile (v/v), 2 % methanol (v/v), pH 5.2] was 350 or 450 µl/min, respectively in a step gradient application. The enrichment and stripping flow rate of buffer [10 mM potassium phosphate, pH 5.2] was during 4 min and the total runtime was 55 min. The HPLC system used was a Shimadzu LC-20 AD Analytical & Measuring Instruments System, with an Agilent 1100 Series Variable Wavelength Detector set at 253nm. Concentrations were calculated by a two-point calibration curve using internal standard method. The data are expressed as pmol per mg protein or pmol per mL.

Immunohistochemical staining for NTPDase1

After the fixative was washed out, the coronal brain sections were incubated in blocking solution (5% normal goat serum and 1 mg/ml BSA) for 1 h at 22 ˚C. Incubation in the solution of the polyclonal NTPDase1 antibody (1:500, #rN1-6L, I4), was performed overnight at 4 ^{o}C. Following three, 10 min washes in PBS, the sections were incubated with biotinylated secondary antibody for 2 h. The staining was performed with Vectastain ABC Elite kit using DAB as the chromogen. After washing thoroughly with distilled water, sections were postfixed in 1% OsO4, dehydrated in ethanol, stained with 1% uranyl acetate in 50% ethanol for 30 min, and embedded in Taab 812. Negative control experiments were performed using the same protocol but substituting pre-immune serum for the primary antibody. Ultrathin sections were cut using a Leica UCT ultramicrotome (Leica Microsystems, Milton Keynes, UK) and examined using a Hitachi 7100 transmission electron microscope (Hitachi; Tokyo, Japan).

Enzyme histochemistry for detection of ecto-ATPase activity

A cerium precipitation method was used for electron microscopic investigation of ecto-ATPase activity[14]. Briefly, thoroughly washed sections were incubated in a medium containing 1 mM ATP as substrate, 3 mM CeCl3 (precipitating agent for the liberated phosphate), 1 mM levamisole (inhibitor of alkaline phosphatases, Amersham, Poole, UK), 1

mM ouabain (Na+, K+-ATPase inhibitor; Merck, Darmstadt, Germany), 50 mM αβ-methylene ADP (5'-nucleotidase inhibitor) and 5 mM KCl in 70 mM Tris–maleate buffer (pH 7.4) for 30 min at room temperature. Incubation was followed by three rinses in Tris–maleate buffer and washing with distilled water. The tissue blocks were then postfixed, dehydrated and treated and embedded into Taab 812 resin for ultrathin sectioning and microscopic examination as described above. Control reactions were performed without adding the ATP substrate.

Flow cytometric analysis of brain, spleen and blood samples

Cells were isolated from mouse brains by enzymatic digestion with the mixture of DNase I (10µg/ml, #11284932001, Roche) and Collagenase/Dispase (0,5mg/ml, #11088866001, Roche) in 10% FCS/DMEM, followed by several centrifugation steps with Percoll (40% and 70%) and washing in 10% FCS/DMEM. Spleen cells were isolated by mechanical homogenization of the spleen and red blood cells were removed by centrifugation. Brain and spleen cells were diluted with FACS buffer (PBS containing 0.1% Tween 20) before acquisition. Venous blood was collected from the heart before transcardial perfusion using 3.8% sodiumcitrate as an anticoagulant. For flow cytometric analysis, Fc receptor blockade was performed (anti-mouse CD16/CD32, 1:100, #16-0161-85, eBioScience), followed by labelling blood cells, splenic leukocytes or brain cells with cocktails of selected antibodies: anti-mouse CD8a-PE (1:400, #12-0081-82 eBioScience); anti-mouse CD3e-APC (1:200, #17-0032-80, eBioScience), anti-mouse CD11b-PE (1:200, #101207, BioLegend); anti-mouse Ly-6C-PE-Cy7 (1:400, #25-5932-80, eBioScience); anti-mouse CD115-APC (1:100, # 17-1152-80, eBioScience), anti-mouse F4/80-like receptor-BV421 (1:200, #563900, BD Biosciences), anti-mouse CD39-PE (1:100, #143803, BioLegend), CD45-PercyP5.5 (1:200, #103131, BioLegend) and for viability Zombie Violet (1:200, #423113, BioLegend). Cells were acquired on a BD FACSVerse flow cytometer and data were analysed using FACSuite software (BD Biosciences). Total blood cell counts were calculated by using 15 µm polystyrene microbeads (#18328-5, Polysciences).

References:

1 Barna L, Dudok B, Miczan V, Horvath A, Laszlo ZI, Katona I (2016) Correlated confocal and super-resolution imaging by VividSTORM. Nat Protoc 11: 163-183 Doi 10.1038/nprot.2016.002

2 Boldogkoi Z, Balint K, Awatramani GB, Balya D, Busskamp V, Viney TJ, Lagali PS, Duebel J, Pasti E, Tombacz Det al (2009) Genetically timed, activity-sensor and rainbow transsynaptic viral tools. Nat Methods 6: 127-130 Doi 10.1038/nmeth.1292

3 Boldogkoi Z, Reichart A, Toth IE, Sik A, Erdelyi F, Medveczky I, Llorens-Cortes C, Palkovits M, Lenkei Z (2002) Construction of recombinant pseudorabies viruses optimized for labeling and neurochemical characterization of neural circuitry. Brain Res Mol Brain Res 109: 105-118

4 Boldogkoi Z, Sik A, Denes A, Reichart A, Toldi J, Gerendai I, Kovacs KJ, Palkovits M (2004) Novel tracing paradigms--genetically engineered herpesviruses as tools for mapping functional circuits within the CNS: present status and future prospects. Prog Neurobiol 72: 417-445 Doi 10.1016/j.pneurobio.2004.03.010

5 Chiovini B, Turi GF, Katona G, Kaszas A, Palfi D, Maak P, Szalay G, Szabo MF, Szabo G, Szadai Zet al (2014) Dendritic spikes induce ripples in parvalbumin interneurons during hippocampal sharp waves. Neuron 82: 908-924 Doi 10.1016/j.neuron.2014.04.004

6 Czondor K, Ellwanger K, Fuchs YF, Lutz S, Gulyas M, Mansuy IM, Hausser A, Pfizenmaier K, Schlett K (2009) Protein kinase D controls the integrity of Golgi apparatus and the maintenance of dendritic arborization in hippocampal neurons. Mol Biol Cell 20: 2108-2120 Doi 10.1091/mbc.E08-09-0957

7 Denes A, Boldogkoi Z, Hornyak A, Palkovits M, Kovacs KJ (2006) Attenuated pseudorabies virus-evoked rapid innate immune response in the rat brain. J Neuroimmunol 180: 88-103 Doi 10.1016/j.jneuroim.2006.07.008

8 Denes A, Coutts G, Lenart N, Cruickshank SM, Pelegrin P, Skinner J, Rothwell N, Allan SM, Brough D (2015) AIM2 and NLRC4 inflammasomes contribute with ASC to acute brain injury independently of NLRP3. Proc Natl Acad Sci U S A 112: 4050-4055 Doi 10.1073/pnas.1419090112

9 Dudok B, Barna L, Ledri M, Szabo SI, Szabadits E, Pinter B, Woodhams SG, Henstridge CM, Balla GY, Nyilas Ret al (2015) Cell-specific STORM super-resolution imaging reveals nanoscale organization of cannabinoid signaling. Nat Neurosci 18: 75-86 Doi 10.1038/nn.3892

10 Elmore MR, Najafi AR, Koike MA, Dagher NN, Spangenberg EE, Rice RA, Kitazawa M, Matusow B, Nguyen H, West BLet al (2014) Colony-stimulating factor 1 receptor signaling is necessary for microglia viability, unmasking a microglia progenitor cell in the adult brain. Neuron 82: 380-397 Doi 10.1016/j.neuron.2014.02.040

11 Gonci B, Nemeth V, Balogh E, Szabo B, Denes A, Kornyei Z, Vicsek T (2010) Viral epidemics in a cell culture: novel high resolution data and their interpretation by a percolation theory based model. PLoS One 5: e15571 Doi 10.1371/journal.pone.0015571

12 Horvath G, Goloncser F, Csolle C, Kiraly K, Ando RD, Baranyi M, Kovanyi B, Mate Z, Hoffmann K, Algaier Iet al (2014) Central P2Y12 receptor blockade alleviates inflammatory and neuropathic pain and cytokine production in rodents. Neurobiol Dis 70: 162-178 Doi 10.1016/j.nbd.2014.06.011

13 Katona G, Szalay G, Maak P, Kaszas A, Veress M, Hillier D, Chiovini B, Vizi ES, Roska B, Rozsa B (2012) Fast two-photon in vivo imaging with three-dimensional random-access scanning in large tissue volumes. Nat Methods 9: 201-208 Doi 10.1038/nmeth.1851

14 Kittel A (1999) Lipopolysaccharide treatment modifies pH- and cation-dependent ecto-ATPase activity of endothelial cells. J Histochem Cytochem 47: 393-400 Doi 10.1177/002215549904700313

15 Kornyei Z, Szlavik V, Szabo B, Gocza E, Czirok A, Madarasz E (2005) Humoral and contact interactions in astroglia/stem cell co-cultures in the course of glia-induced neurogenesis. Glia 49: 430-444 Doi 10.1002/glia.20123

16 Kremer JR, Mastronarde DN, McIntosh JR (1996) Computer visualization of three-dimensional image data using IMOD. J Struct Biol 116: 71-76 Doi 10.1006/jsbi.1996.0013

17 Szalay G, Martinecz B, Lenart N, Kornyei Z, Orsolits B, Judak L, Csaszar E, Fekete R, West BL, Katona Get al (2016) Microglia protect against brain injury and their selective elimination dysregulates neuronal network activity after stroke. Nat Commun 7: 11499 Doi 10.1038/ncomms11499
